# Regulation of mouse exploratory behaviour by irradiance and cone-opponent signals

**DOI:** 10.1186/s12915-023-01663-6

**Published:** 2023-08-21

**Authors:** E. Tamayo, J. W. Mouland, R. J. Lucas, T. M. Brown

**Affiliations:** Centre for Biological Timing, Faculty of Biology, Medicine and Health, University of Manchester, Manchester, UK

**Keywords:** Photoreceptor, Masking, Hypothalamus, Mouse, Behaviour, Electrophysiology, Cone, Melanopsin

## Abstract

**Background:**

Animal survival depends on the ability to adjust behaviour according to environmental conditions. The circadian system plays a key role in this capability, with diel changes in the quantity (irradiance) and spectral content (‘colour’) of ambient illumination providing signals of time-of-day that regulate the timing of rest and activity. Light also exerts much more immediate effects on behaviour, however, that are equally important in shaping daily activity patterns. Hence, nocturnal mammals will actively avoid light and dramatically reduce their activity when light cannot be avoided. The sensory mechanisms underlying these acute effects of light are incompletely understood, particularly the importance of colour.

**Results:**

To define sensory mechanisms controlling mouse behaviour, we used photoreceptor-isolating stimuli and mice with altered cone spectral sensitivity (*Opn1mwR*), lacking melanopsin (*Opn1mwR*; *Opn4*^*−/−*^) or cone phototransduction (*Cnga3*^*−/−*^) in assays of light-avoidance and activity suppression. In addition to roles for melanopsin-dependent irradiance signals, we find a major influence of spectral content in both cases. Hence, remarkably, selective increases in S-cone irradiance (producing a blue-shift in spectrum replicating twilight) drive light-seeking behaviour and promote activity. These effects are opposed by signals from longer-wavelength sensitive cones, indicating a true spectrally-opponent mechanism. Using c-Fos-mapping and multielectrode electrophysiology, we further show these effects are associated with a selective cone-opponent modulation of neural activity in the key brain site implicated in acute effects of light on behaviour, the subparaventricular zone.

**Conclusions:**

Collectively, these data reveal a mechanism whereby blue-shifts in the spectrum of environmental illumination, such as during twilight, promote mouse exploratory behaviour.

**Supplementary Information:**

The online version contains supplementary material available at 10.1186/s12915-023-01663-6.

## Background

Environmental light constitutes a major influence on animal behaviour. On one hand, light is a key regulator of the circadian system, which in turn drives daily rhythms in rest and activity [1]. Alongside this, however, light can rapidly and acutely influence behaviour. For example, nocturnal mammals will actively avoid brightly illuminated areas if possible or will dramatically reduce their behavioural activity and rest when bright light cannot be avoided [2–4]. Alongside circadian control mechanisms, such acute effects of light on behaviour therefore constitute a major determinant of the daily patterns of mammalian rest and activity [4, 5].

Given their importance for regulating animal behaviour, significant past activity has attempted to define the sensory mechanisms regulating both the circadian system and acute effects of light on activity. A key development here was the identification of a retinal cell type specialised for encoding ambient light levels—intrinsically photosensitive retinal ganglion cells (ipRGCs)—and the subsequent realisation that both circadian and many acute effects of light on behaviour are lost in animals lacking ipRGCs [6–9]. Nonetheless, the photoreceptive mechanisms underlying the effects of light on behaviour have continued to prove challenging to define since ipRGCs are known to combine intrinsic, melanopsin-dependent, excitation with synaptically mediated signals originating with rods and cones [10]. Moreover, we now know of multiple subtypes of ipRGC with differing properties [11].

In accordance with this potential complexity in the sensory properties of ipRGC-mediated responses, studies of the photoreceptor mechanisms regulating the mouse circadian system provide evidence for two distinct sources of control [1]. Hence, in addition to irradiance signals (derived from a combination of rod and melanopsin photoreception; [12–14]), mouse circadian responses are modulated according to the spectral content (‘colour’) of ambient illumination by opponent influences of short and longer wavelength sensitive cone opsins (equivalent to the blue-yellow axis of human colour vision) [15, 16].

By contrast to the above, understanding of the sensory mechanisms controlling acute effects of light on rodent behaviour (light avoidance/aversion and suppression of activity) is currently less complete. Such effects have been most extensively studied in the context of the ability of bright light to suppress rodent behavioural activity (typically measured by voluntary wheel running in the animals’ home cage)—a response commonly referred to as ‘negative masking’ [4]. The observations that melanopsin knockout mice show substantially reduced negative masking and that mice lacking rods and cones do not show any noticeable reduction in the sensitivity or amplitude of such responses [17–21] collectively establish melanopsin as a major regulator of these irradiance-dependent effects on behaviour. Nonetheless, the partial retention of negative masking in melanopsin knockout mice [18, 19], similarly suggests a role for one or more outer retinal photoreceptor classes, as does one of two studies that evaluated the spectral sensitivity of such responses in wildtype mice [20, 22]. Similarly for studies of light avoidance responses outside the home-cage environment, aside from neonatal animals (where such responses appear to be fully-dependent on melanopsin; [23, 24]), existing data provides evidence that both melanopsin and rod/cone signals contribute to such responses [8, 9, 25, 26]., In sum, while a contribution of melanopsin to both light avoidance and light-induced suppression of activity (negative masking) is well established, the specific contribution of the two mouse cone types and/or the possibility of any colour-dependent influences on such behaviours remains essentially un-investigated.

Here, then, we set out to define the sensory mechanisms regulating light avoidance behaviour and light-induced suppression of activity in mice. To date, efforts to define the underlying photoreceptor control mechanisms have been hampered by the strongly overlapping spectral sensitivities of melanopsin, rhodopsin and M-cone opsin in mice. Here we overcome those issues by using mice where the native mouse M-cone opsin is replaced by the human L-cone opsin (*Opn1mw*^*R*^; [27]), alongside photoreceptor isolating stimuli that allow us to manipulate excitation of the two mouse cone opsin classes independently of each other (and melanopsin and rods)—an approach that has produced powerful insight into many other aspects of mouse vision [14, 15, 28–31]. Accordingly, we here now provide evidence for a significant spectrally-opponent modulation of both light avoidance and light-induced suppression of activity in mice, as well as confirming an equivalent cone-opponent modulation of neural activity in a key brain node implicated in regulating acute effects of light on behaviour—the hypothalamic supraventricular zone.

## Results

### Factors influencing light–dark preference in mice

**Fig. 1:**
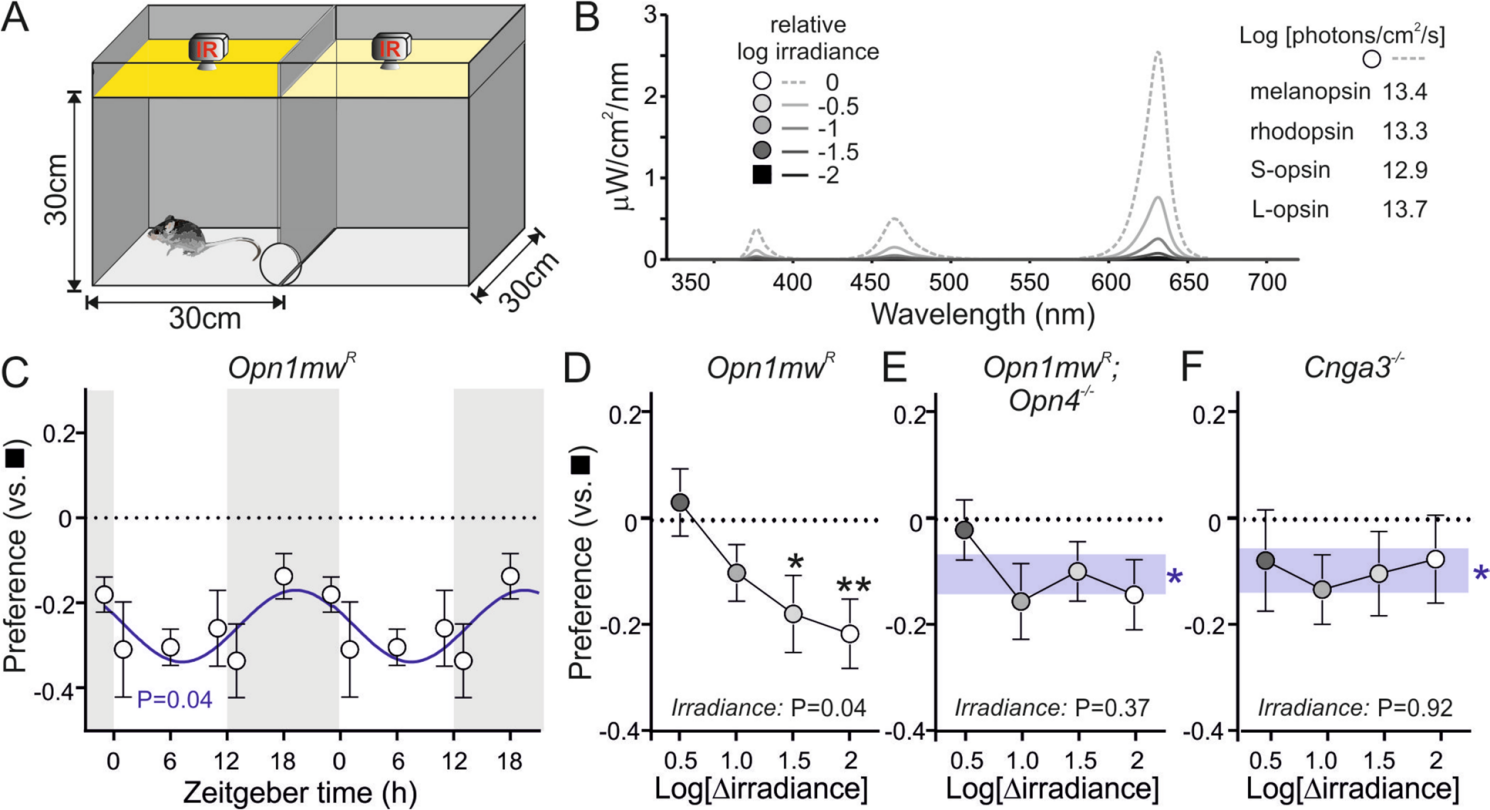
Irradiance-dependent light avoidance requires melanopsin and cone photoreception.** A** Schematic of the apparatus for assessing light:dark preference, comprising two interconnected and independently illuminated chambers. **B** Spectral composition of initial test stimuli, which recreated a wildtype mouse’s experience of natural daylight over a 2-order of magnitude range. **C** Mean ± SEM preference index ([*T*_Bright_ − *T*_Dim_]/[*T*_Bright_ + *T*_Dim_]) for *Opn1mw*^*R*^ mice (*n* = 6) given a choice between the brightest vs dimmest test stimuli, double plotted as a function of time of testing (stimuli randomised across left and right sides of the chamber within and between animals). Data analysed by comparison of sinusoidal fit vs. null hypothesis of zero slopes first order polynomial fit (*F*-test—*F*_1,34_ = 4.43; *P* = 0.043). **D**–**F** Mean ± SEM preference index (testing at ZT 4.5–7.5) for *Opn1mw*^*R*^ (**D**; *n* = 16), *Opn1mw*^*R*^; *Opn4*^*−/−*^ (**E**; *n* = 11) and *Cnga3*^*−/−*^ mice (**F**, *n* = 12), when comparing varying ‘bright’ irradiance against the dimmest test stimulus. Data analysed by one-way RM ANOVA (**D**: *F*_2.852, 42.78_ = 3.11, *P* = 0.039; **E**: *F*_2.651, 26.51_ = 1.08, *P* = 0.37; **F**: *F*_2.262, 24.89_ = 0.109, *P* = 0.92) with one-sample *t*-tests vs. a hull hypothesis of 0 preference, as appropriate. Shaded region in **E** and **F** represents mean ± SEM ‘bright’ preference across tested irradiances, with one sample *t*-tests vs. a hull hypothesis of 0 preference. * and ** represent *P* < 0.05 and *P* < 0.01, respectively

To provide insight into the photoreceptive mechanisms influencing mouse exploratory activity, we first established a modified version of conventional light–dark preference test [2] whereby, on entry to the apparatus, mice could move freely between two independently illuminated chambers, with behavioural monitoring via overhead IR cameras (Fig. 1A). Diffuse illumination was supplied by a 4-primary LED-based system allowing us to freely adjust intensity and spectral composition across the two chambers, enabling within-animal assessments of the role of specific photoreceptor classes in light-avoidance.

We initially confirmed that the expected innate aversion to light was apparent in *Opn1mw*^*R*^ mice (*n* = 6) under our experimental conditions and asked whether this preference differed as a function of time of testing. To this end, we first established an illumination setting that recreated the relative pattern of photoreceptor activation consistent with a wildtype mouse’s experience of an overcast day [16] and then provided mice with a choice between bright and 100-fold dimmer versions of this stimulus (Fig. 1B). Across a cohort of six *Opn1mw*^*R*^ mice tested over 6 timepoints spanning their homecage LD cycle, as expected, we observed a consistent avoidance of the ‘bright’, and preference towards the ‘dim’, side of the apparatus (Mean ± SEM ‘bright’ preference index =  − 0.26 ± 0.04, one sample *t*-test, *P* = 0.002). Interestingly, however, we also found a modest, but significant, diurnal variation in preference, with reduced light avoidance during testing at night (Fig. 1C). We also found that this light avoidance behaviour was associated with a more fine-grained changes in mouse behaviour. Hence, when in the bright side of the apparatus, *Opn1mw*^*R*^ mice consistently spent more of their time in the corners of the chamber when compared to when in the dim side (Mean ± SEM: 78.9 ± 0.9% vs. 69.6 ± 1.1%, paired *t*-test, *P* < 0.001; Additional file 1: Fig. S1A), a behaviour often considered as an indicator of heightened anxiety [32]. This attraction to the corners of the chamber under high light levels did not vary as a function of time of testing while, interestingly, behaviour in the dark side of the chamber exhibited a diurnal rhythm with greater time in the corners during the day. Furthermore, we found that the total distance travelled by mice was significantly reduced for the bright vs. dim side of the apparatus (mean ± SEM: 16.5 ± 0.8 m vs. 22.4 ± 0.8 m, paired *t*-test, *P* < 0.001; Additional file 1: Fig. S1E). In this case, however, we found a modest diurnal rhythm when on the bright side, with reduced distance travelled during the midday vs. night, but no significant variation in distance travelled on the dim side of the apparatus. Together these data indicate that the midday epoch was also associated with the most robust light-avoidance responses. Accordingly, we focused subsequent testing during this portion of the day (ZT 4.5–7.5).

We next assessed the extent to which light avoidance was impacted by irradiance. To this end, we tested *Opn1mw*^*R*^ mice (*n* = 16) under conditions where the dim side of the chamber was fixed as in the preceding experiment and the bright side of the chamber was between 0.5 and 2 log units brighter. Under these conditions we found light avoidance scaled with the irradiance of the bright side, with the most robust responses occurring when this was > 1 log unit brighter than the dim side (Fig. 1D). Given the intensity range under which this effect was observed (extending above the point where rods provide useful information about irradiance [33–35] or support robust visually-guided behaviour [36–38]), and previous data on photoreceptor contributions to light avoidance [8, 9, 25], we reasoned that cones and/or melanopsin signals may be particularly important in influencing the animal’s preference.

To provide insight into these photoreceptor contributions, we then performed equivalent experiments in animals lacking melanopsin (*Opn1mw*^*R*^; *Opn4*^*−/−*^; *n* = 11, Fig. 1E) or cone phototransduction (*Cnga3*^*−/−*^; *n* = 12, Fig. 1F). Interestingly, while both groups of animals retained a clear general preference to dim across the test comparisons (Mean ± SEM ‘bright’ preference index =  − 0.10 ± 0.03 and − 0.10 ± 0.04 for *Opn1mw*^*R*^; *Opn4*^*−/−*^ and *Cnga3*^*−/−*^ respectively, one-sample *t*-tests, both *P* < 0.05) in neither case could we detect a significant irradiance dependence to the response (Fig. 1E, F). Consistent, then, with previous data suggesting both inner and outer retinal photoreceptors can influence light avoidance [8, 25, 26], these data suggest that both melanopsin and cones contribute to irradiance-dependent changes in exploratory behaviour under our experimental conditions. In line with our previous data, all three groups of animals also tended to spend a greater proportion of time in the corners of the test chambers (Additional file 1: Fig. S1B–D) and travelled less overall distance (Additional file 1: Fig. S1F–H) under brighter vs. dim conditions. In agreement with the changes in preference described above, *Opn1mw*^*R*^ displayed an irradiance-dependent reduction in total distance travelled on the brighter side of the test apparatus (Additional file 1: Fig. S1F), whereas this irradiance dependence was lost in *Opn1mw*^*R*^; *Opn4*^*−/−*^ and *Cnga3*^*−/−*^ mice (Additional file 1: Fig. S1F,H). By contrast, we found that irradiance influenced the time spent in corners of the test apparatus for both *Opn1mw*^*R*^ and *Cnga3*^*−/−*^ mice (Additional file 1: Fig. S1B and D) but not in *Opn1mw*^*R*^; *Opn4*^*−/−*^ animals (Additional file 1: Fig. S1C). This suggests an especially important role for melanopsin in driving this particular feature of light-dependent mouse behaviour, consistent with previous work showing melanopsin is a key driver of similar responses to light in other assays [39].

### Cone contributions to mouse exploratory behaviour

**Fig. 2:**
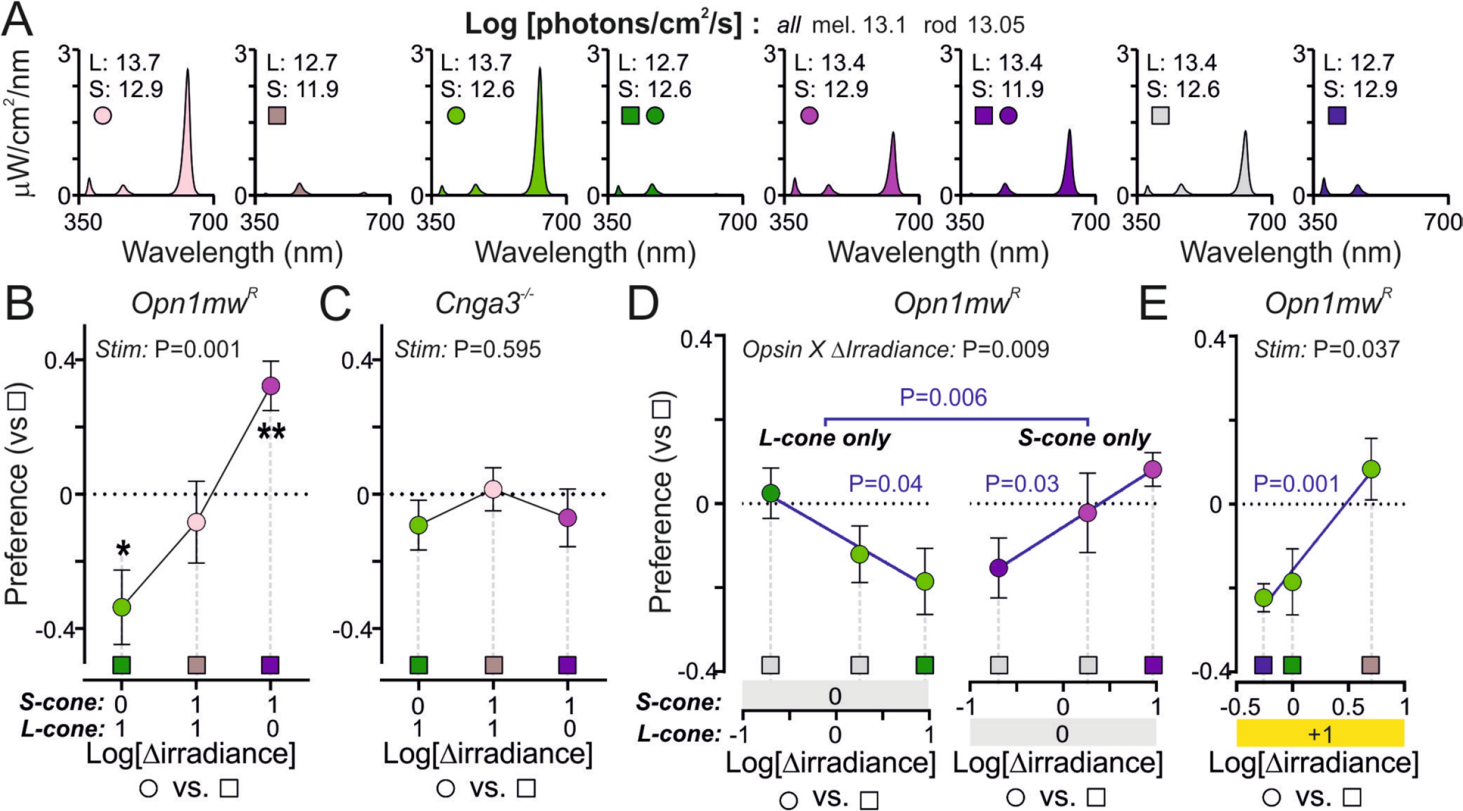
Cone-opponent colour signals modulate light avoidance behaviour in mice. (**A**) Spectral power distributions of test stimuli matched in irradiance for melanopsin and rods but differed in irradiance for S- and/or L-cone opsin. **B**, **C** Mean ± SEM preference of *Opn1mw*^*R*^ (**B**; *n* = 7), and *Cnga3*^*−/−*^ mice (**C**, *n* = 13) to L- and/or S-cone ‘bright’ (○) vs. ‘dim’ (□) lighting conditions. Data analysed by one-way RM ANOVA (**B**: *F*_2, 18_ = 9.87, *P* = 0.001; **C**: *F*_2, 36_ = 0.53, *P* = 0.595) with one sample *t*-tests vs. null hypothesis of 0 preference as appropriate. (**D**) Mean ± SEM preference of *Opn1mw*^*R*^ mice (*n* = 8) to stimuli represented by (○) as a function of irradiance difference relative to opposing side of chamber (□), where irradiance differed only for L- (left) or S-cone opsin (right). Data analysed by two-way RM ANOVA (opsin—*F*_1, 14_ = 0.947, *P* = 0.347; irradiance—*F*_2, 28_ = 0.043, *P* = 0.959; interaction—*F*_2, 28_ = 5.69, *P* = 0.009). Linear fits tested for non-zero slope (Left- *F*_1,22_ = 4.96, *P* = 0.037; right- *F*_1,22_ = 5.51, *P* = 0.028) and for difference in slopes (left vs. right—*F*_2, 44_ = 5.84, *P* = 0.006) via *F*-test. **E** Mean ± SEM preference of *Opn1mw*.^*R*^ mice (*n* = 8) to stimuli represented by (○), which always presented a 1 log unit difference in irradiance for L-cones compared to the opposing side of chamber (□) but a variable difference in irradiance for S-cones. Data analysed by one-way RM ANOVA (*F*_1.393, 9.751_ = 5.28, *P* = 0.037). Linear fit tested for non-zero slope via *F*-test (*F*_1, 22_ = 13.33, *P* = 0.001)

Given our data suggesting that cone signals influence the choice of mice to explore bright vs. dim environments, we next sought to define how the different classes of cone photoreceptors might contribute to such behavioural decisions. To this end, we designed a range of illumination settings which had identical brightness for rods and melanopsin but differing brightness for L- and/or S-cone opsin (Fig. 2A). We then started by assessing the preference of *Opn1mw*^*R*^ mice (*n* = 7) under conditions where the two sides of the chamber selectively differed in brightness by an order of magnitude for both L- and S-opsin (Fig. 2B). Surprisingly, we found that mice did not show a consistent preference towards ‘bright’ or ‘dim’ sides of the chamber under these conditions (nor did they show a difference in total distance travelled; Additional file 1: Fig. S2A). By contrast, when the two sides of the chamber selectively differed in brightness for just L- or S-cone opsin the same mice exhibited clear preferences that were opposite in polarity for the two comparisons (Fig. 2B). Hence, when there was a selective difference in brightness for L-cone opsin, mice exhibited the expected preference towards the ‘dim’ side of the chamber but, remarkably, choose the ‘bright’ side of the chamber when irradiance only differed for S-cone opsin. These effects were accompanied by reductions in total distance travelled on the L-cone ‘bright’ and S-cone ‘dim’ sides of the chamber (Additional file 1: Fig. S2A). Collectively then these data reveal a cone-opponent mechanism that modulates the natural tendency of mice to favour more dimly illuminated environments whereby they prefer environments that are relatively enriched for S- vs. L-cone opsin stimulation, akin to the blue-shifted spectra that mice and other mammals experience during twilight [15, 16, 40].

To confirm that the data above reflected a genuine role of cones we next repeated the same experiment in *Cnga3*^*−/−*^ mice (*n* = 13). As expected in these animals lacking cone phototransduction, these was no consistent preference observable for any of the test stimuli (Fig. 2C), nor was there any observable effect on total distance travelled (Additional file 1: Fig. S2B). Accordingly, having confirmed that the impact of our test conditions in *Opn1mw*^*R*^ mice could not simply be explained by some unintended difference in brightness for rods and melanopsin, we next set out to better understand the nature of the cone-mediated effect.

To dissect out which features of the spectral choices we presented were influencing preference, in a separate cohort of *Opn1mw*^*R*^ mice (*n* = 8), we assessed preference to the same L- and S-opsin ‘bright’ and ‘dim’ stimulus conditions when compared against each other and when paired with a neutral background stimulus (Fig. 2D). Since the latter was matched to all the other test stimuli in irradiance for melanopsin, rods (and as appropriate L- or S-opsin), this allowed us to present mice with a range of selective difference in brightness for just L- or S-cone opsin. These experiments revealed that the preference exhibited by the mice was dictated by differences in L- or S-cone irradiance across the two sides of the chamber rather than a particular preference towards or aversion from specific elements of the test stimuli employed. Moreover, consistent with our data above, the slopes of these relationships were opposite such that preference was negatively correlated with the difference in L-opsin irradiance (i.e. increased light avoidance) but positively correlated with the difference in S-opsin irradiance (Fig. 2D). As expected, these effects were also accompanied by differences in total distance travelled by mice, with increased differences in L-opsin irradiance decreasing distance travelled and increased differences in S-opsin irradiance driving the opposite relationship (Additional file 1: Fig. S2C).

Given the findings above (Fig. 2B, D), we hypothesised that signals from both cone types should combine in an antagonistic (opponent) manner to dictate a mouse’s preference towards a specific environment. Hence, a mouse’s aversion to L-cone ‘bright’ vs. ‘dim’ environments should be respectively reduced or enhanced with increasing or decreasing irradiance for S-cones. To test this hypothesis, we next compared conditions where *Opn1mw*^*R*^ mice (*n* = 8, same cohort as in Fig. 2D) were consistently presented with a choice between chambers that differed in L-opsin irradiance by an order of magnitude but where the difference in irradiance for S-opsin varied between − 0.3 and + 0.7 log units. As expected, avoidance of (and distance travelled under) the L-opsin ‘bright’ chamber varied substantially as a function of the concomitant difference for S-opsin (Fig. 2E, Additional file 1: Fig. S2D). Hence, when the L-opsin ‘dim’ chamber had comparatively high irradiance for S-opsin (and was therefore comparatively ‘blue’) aversion to ‘bright’/preference to ‘dim’ was maximised whereas when the L-opsin ‘dim’ chamber had very low irradiance for S-opsin (more ‘yellow’), aversion to the L-opsin ‘bright’ chamber disappeared. In sum, these data confirm that signals from the two cone types combine in an opponent manner so as to promote mouse exploration of environments where the spectral content of ambient illumination more resembles the blue-shift occurring during twilight.

### Cone influences on light-induced suppression of activity

**Fig. 3:**
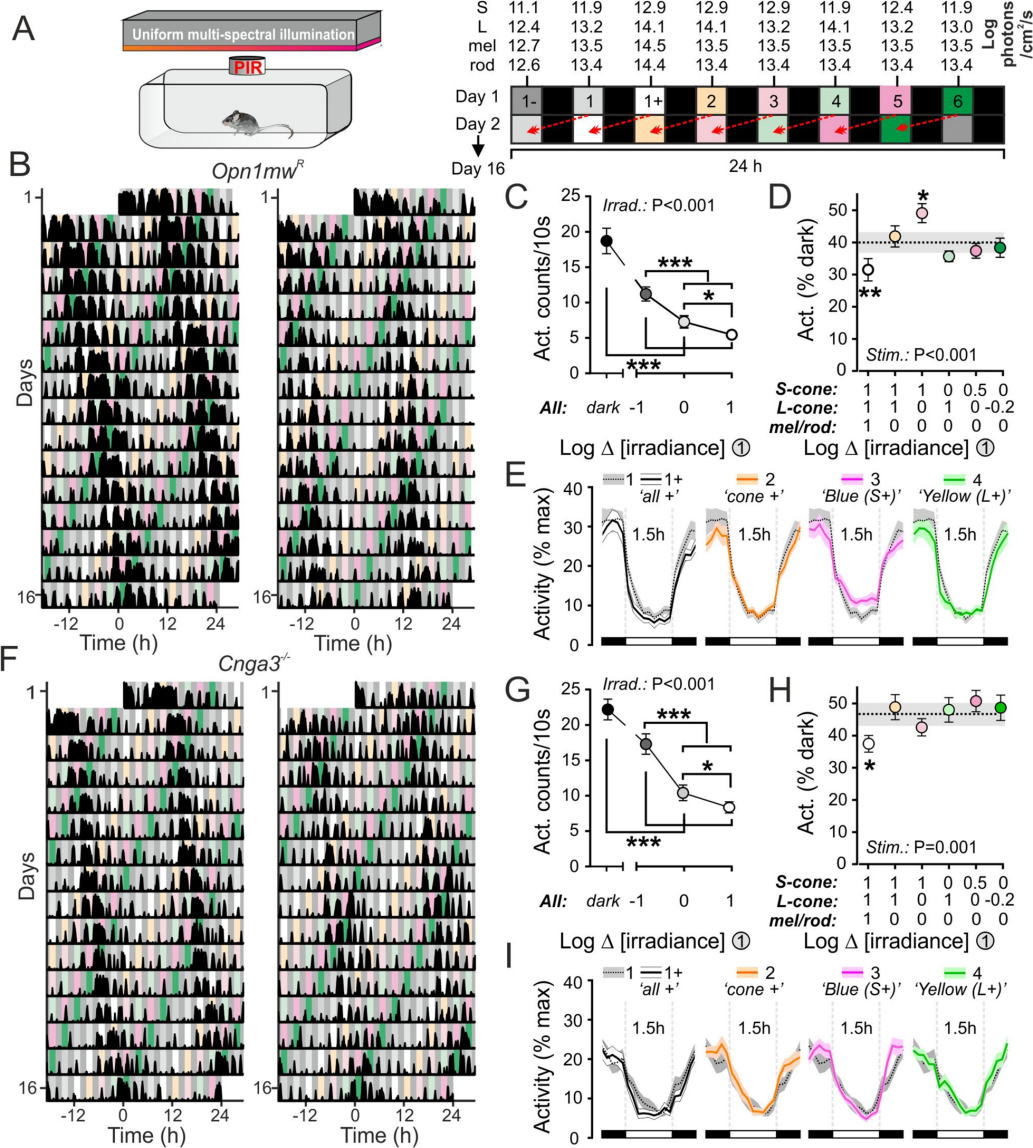
S-cone opponent colour signals modulate light-induced masking. **A** Experimental setup for passive infrared (PIR) activity monitoring and paradigm for delivery of stimuli of varying spectral composition (See Additional file 1. Fig. S3 for spectra). **B**, **F** Example PIR activity records from two *Opn1mw*^*R*^ (**B**) and *Cnga3*^*−/−*^ (**F**) mice. **C**, **G** Mean ± SEM activity counts during dark at varying intensities of ‘spectra 1’ for *Opn1mw*^*R*^ (**C**, *n* = 12) and *Cnga3*^*−/−*^ (**G**, *n* = 12) mice. Data analysed by one-way RM ANOVA with Tukey’s post tests (**C**: *F*_1.2,12.9_ = 62.4, *P* < 0.001; **G**: *F*_2.0,21.7_ = 107.3, *P* < 0.001). **D**, **H** Mean ± SEM activity suppression across test stimuli for *Opn1mw*^*R*^ (**D**, *n* = 12) and *Cnga3*^*−/−*^ (**H**, *n* = 12) mice. Shaded region indicates mean ± SEM activity suppression for the reference stimulus (‘spectra 1’). Data analysed by one-way RM ANOVA with Sidak’s post-tests against reference spectra (**D**: *F*_4.0,43.8_ = 8.0, *P* < 0.001; **H**: *F*_4.1,44.6_ = 5.3, *P* = 0.001). **E**, **I** Mean ± SEM normalised activity for *Opn1mw*^*R*^ (**E**, *n* = 12) and *Cnga3*.^*−/−*^ (**I**, *n* = 12) mice across the 1.5-h light phase for spectra 1 + , 2, 3 and 4 and comparison to the reference spectra (spectra 1)

We next set out to determine whether the opponent impact of cone signals identified above was specific to environmental preference outside of the home cage environment or reflected a more global modulation of exploratory activity. To this end, we housed mice in cabinets which allowed for dynamic modulation of the intensity and spectral composition of environmental illumination and assessed the impact of a range of different lighting conditions on passive infrared (PIR)-measured activity (Fig. 3A). Specifically, we established a range of stimuli that either provided global (spectrally neutral) differences in irradiance relative to a reference spectra (‘spectra 1’) or selectively differed in brightness for L- and/or S-cone opsins (Additional file 1: Fig. S3A). We then presented the test stimuli as an alternating interleaved 1.5 h:1.5 h LD cycle (Fig. 3A). As expected based on past studies that have used related ultradian light cycles [41, 42], under these conditions, *Opn1mw*^*R*^ mice (*n* = 12) retained significant circadian rhythms in behaviour with a relatively long free-running period (Mean ± SEM = 24.7 ± 0.14 h; Fig. 3B; Additional file 1: Fig. S3B,C). As a result, our experimental paradigm ensured that the presentation of each stimulus was equally distributed across circadian phases of the mouse’s behavioural activity over 16 days of monitoring (Fig. 3B).

We first examined the average activity levels of these 12 *Opn1mw*^*R*^ mice, tested as above, during epochs of darkness and during presentations of varying intensities of the reference spectra, spanning a 1.8 log unit range (equivalent to light intensities encountered across civil twilight to sunset/sunrise; Additional file 1: Fig. S3F). As expected, *Opn1mw*^*R*^ activity levels reliably decreased in an intensity-dependent manner (Fig. 3C), indicating robust light-induced activity suppression (often termed ‘negative masking’). We next compared the degree of activity suppression induced by the reference spectra with the other test stimuli to provide insight into the photoreceptor mechanisms underlying the negative masking response. Of note, whereas the stimulus providing a 1 log unit increase in brightness targeting all photoreceptors (‘spectra 1 + ’) reduced activity significantly more than the reference spectra, when this 1 log unit increase in brightness was restricted to just L- and S-cone opsin (‘spectra 2’) the effect disappeared (Fig. 3D, E). Since the intensity of the reference stimulus (13.4 log rod effective photons/cm2/s) was already an order of magnitude above the point at which rod responses to dark–light transitions should be maximal, the further reduction in activity produced by exposure to ‘spectra1 + ’ is best explained by the increased melanopic irradiance of that stimulus. That conclusion is supported by extensive prior literature demonstrating a substantial contribution of melanopsin to negative masking responses [18–22, 26, 43] as well as the well-documented dynamic ranges for rod and melanopsin responses to dark–light transitions (e.g. [30, 33–35, 44–49]).

Strikingly, however, by comparison to the behaviour observed under the reference spectra, *Opn1mw*^*R*^ mice reliably exhibited increased activity levels (Fig. 3D, E) when presented with an otherwise identical stimulus providing a 1 log unit higher irradiance just for S-cone opsin (‘spectra 3’), thereby providing a colour that mimicked the blue-shift in ambient illumination experienced by mice during twilight [16] (Additional file 1: Fig. S3F,G). Thus, S-cone signals oppose light-induced negative masking responses and instead promote behavioural activity. In combination with the absence of an equivalent effect under lighting conditions with increased brightness for both cone types (‘spectra 2’), collectively these data reveal a spectrally-opponent mechanism whereby L- and S-cone signals respectively promote and inhibit masking responses. Consistent with that interpretation, the presentation of a stimulus selectively enriched for L-cone opsin stimulation (‘spectra 4’) produced a nominally greater degree of masking than the reference stimuli (Fig. 3D, E) although this effect did not attain significance, likely reflecting the fact the reference stimulus was already strongly L-opsin-biased (i.e. comparatively ‘yellow’; Additional file 1: Fig. S3A,F). More modest changes in irradiance targeting S- or L-cones, which retained a substantial overall L-cone bias (more akin to a wildtype mouse’s experience of day) also failed to significantly modulate activity levels relative to the reference spectra (Fig. 3D; ‘Spectra 5/6’). Collectively these data indicate the chromatic control of behavioural activity is particularly relevant for variations in S- vs longer wavelength sensitive cone opsin activation typical of those occurring across daylight-twilight transitions [15, 16].

To confirm that the activity-promoting effect of S-cone enriched lighting identified above did not reflect some unintended off-target effect, we next repeated the same experiments in *Cnga3*^*−/−*^ mice (*n* = 12; Fig. 3F). As expected, *Cnga3*^*−/−*^ mice retained circadian rhythms in behaviour (Fig. 3F, Additional file 1: Fig. S3D,E) and irradiance-dependent reductions in behavioural activity (Fig. 3G) indicating they retained a negative masking response. Notably, however, while 1 log unit increase in brightness targeting all photoreceptors (‘spectra 1 + ’) reduced activity significantly more than the reference spectra (as in *Opn1mw*^*R*^ mice), none of the other test stimuli resulted in a significantly different degree of masking (Fig. 3H, I). Hence, the reduced masking observed for ‘spectra 3’ in *Opn1mw*^*R*^ mice cannot be ascribed to an unintended difference in irradiance for melanopsin or rods. On aggregate then, consistent with data from our previous experiments, we find that lighting which is relatively enriched for S- vs. L-opsin stimulation, so as to recreate the blue-shifted in spectrum associated with natural twilight, specifically promotes behavioural activity.

### Hypothalamic targets associated with cone-opponent influences on behaviour

To provide insight into the neural mechanisms underlying the activity-modulating effects of cone-opponent signals, we next assessed c-Fos expression in the brains of dark-adapted *Opn1mw*^*R*^ mice (two groups of *n* = 6 mice) following 30 min light pulses during the projected night. To identify brain regions whose activity was consistent with a role in the behavioural effects we observed, we choose the two spectrally distinct stimuli that produced the nominally greatest difference in suppression of mouse behavioural activity (Additional file 1: Fig. S3A, spectra 3 and 4). Importantly, for this comparison, both stimuli had identical brightness for rods and melanopsin and they had the same average brightness for cones (i.e. mean of L and S-cone irradiance; 13.05 log10 photons/cm^2^/s) but were respectively weighted to bias irradiance towards either L-cones (‘yellow’) or S-cones (‘Blue’).

**Fig. 4:**
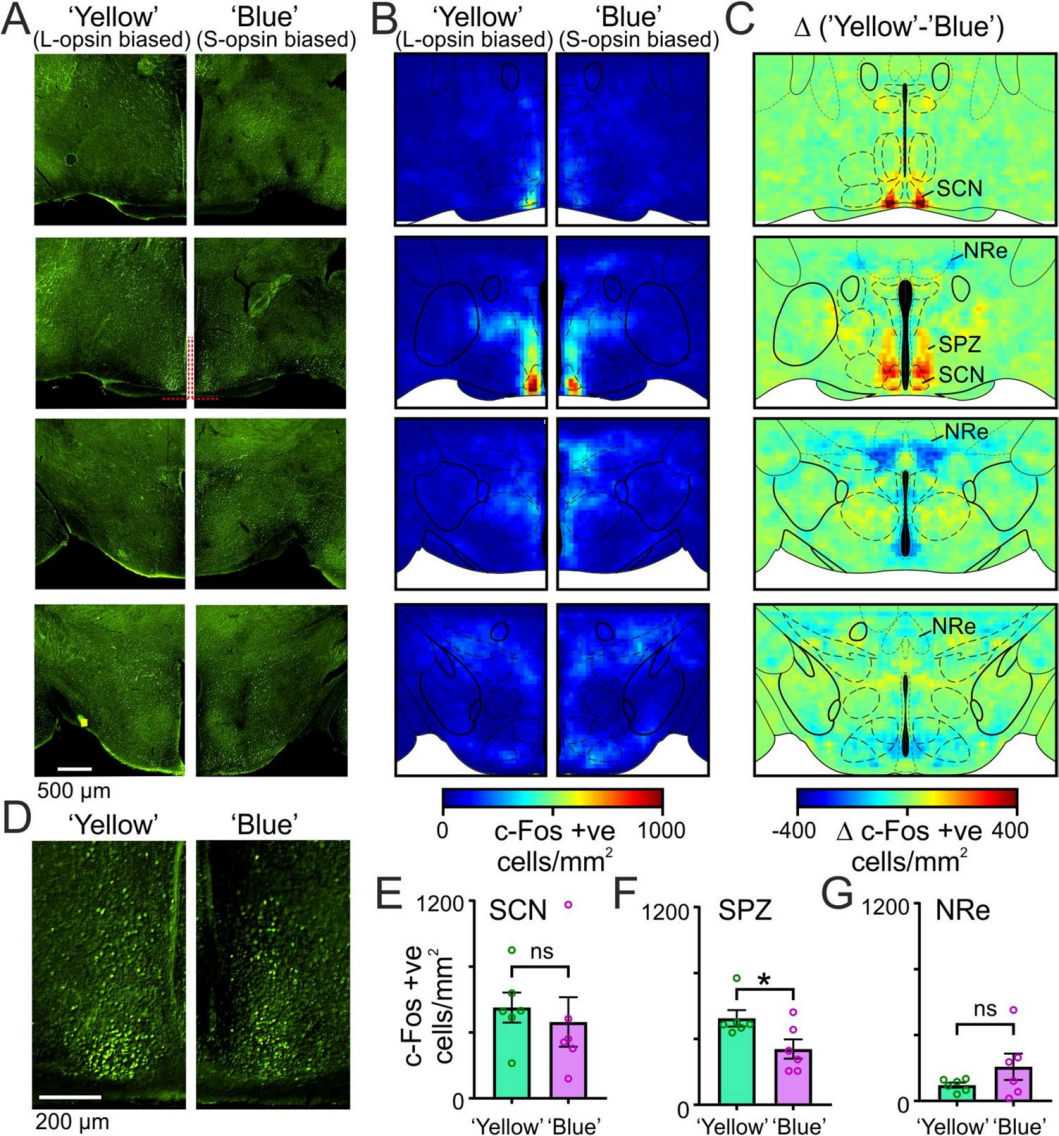
Light steps enriched for long versus short wavelength sensitive cone stimulation differentially activate the ventral supraventricular zone.** A** Representative images of c-Fos immunolabelled hypothalamic hemi-sections from *Opn1mw*.^*R*^ mice following 30 min, rod and melanopsin-isoluminant and cone illuminance matched light pulses biased towards L-opsin (‘Yellow’) or S-opsin (‘Blue’) stimulation (Additional file 1: Fig. S3A, spectra 3 and 4), delivered at projected ZT 17.5. **B** Average maps of c-Fos expression density for ‘Yellow’ and ‘Blue’ light pulses (*n* = 6 mice/group). **C** Spatial map of the mean difference in c-Fos expression density for ‘Yellow’ and ‘Blue’ light pulses (derived from data in **B**). **D** Higher magnification view of c-Fos expression in the SCN and SPZ from **A**. **E**–**G** Mean ± SEM density of c-FOs expression cells in SCN (**E**), SPZ (**F**) and NRe (**G**) for ‘Yellow’ and ‘Blue’ light pulses (*n* = 6/group). Data analyses by unpaired t-test. * = *P* < 0.05, ns = *P* > 0.05

Since the retinohypothalamic tract (RHT) is known to be the primary driver of negative masking responses [4, 50, 51], we first counted c-Fos expressing neurons across portions of the anterior hypothalamus that encompassed RHT target regions [52] (Fig. 4A), to construct average maps of the density of activated neurons for ‘Yellow’ and ‘Blue’ light steps (Fig. 4B, *n* = 6 mice/group). As expected, for both stimuli, these revealed a high density of c-Fos expressing cells in the region of the suprachiasmatic nucleus (SCN) and subparaventricular zone (SPZ), with substantially lower expression densities in surrounding regions of the hypothalamus and ventral thalamus. We next subtracted these spatial maps of average c-Fos expression density to better identify brain regions showing a differential response to the two experimental stimuli (Fig. 4C). This highlighted a subset of brain regions displaying evidence of differential responses, with nominally higher c-Fos expression for the ‘Yellow’ stimulus (which drove greater behavioural activity suppression; Fig. 3D) in the SCN and SPZ and nominally lower expression in ventral portions of the nucleus reuniens (NRe).

Subsequent statistical analysis revealed that the overall density of c-Fos expressing cells in the SCN was equivalent for both experimental stimuli (Fig. 4E; *t*-test, *P* = 0.63). Consistent with data in Fig. 4C, more fine-grained analysis (Additional file 1: Fig. S4A) revealed a tendency towards higher c-Fos expression in rostral and ventral SCN regions for the ‘Yellow’ stimulus but, again, these did not attain significance (*P* = 0.21 and 0.57, respectively). By contrast, c-Fos expression in the ventral SPZ was significantly higher in animals experiencing the ‘Yellow’ vs. ‘Blue’ stimulus (Fig. 4F). This effect was highly specific to the SPZ, since expression patterns for other major hypothalamic nuclei analysed were virtually identical in animals experiencing the two stimuli (Additional file 1: Fig. S4B). Moreover, while our spatial maps suggested the ‘Yellow’ stimulus might also be associated with lower activation of the NRe, that effect also failed to reach statistical significance (Fig. 4G). In summary, then, our data highlight the ventral SPZ (a brain region previously strongly implicated in masking responses [4, 5, 50, 51]) as a potential locus for cone-opponent modulation of mouse activity.

### Cone-opponent regulation of SPZ neural activity

The SPZ is known to receive some direct retinal projections (including from ipRGCs; [52–54]) but is also a major target of efferent projections from the SCN and another retinorecipient region implicated in promoting behavioural activity—the intergeniculate leaflet (IGL) [55, 56]. To confirm the implications of our findings above regarding the existence of cone-opponent regulation of activity and provide more insight into the underlying mechanisms, we performed multielectrode recordings from the SPZ of anaesthetised *Opn1mw*^*R*^ mice (*n* = 4).

**Fig. 5:**
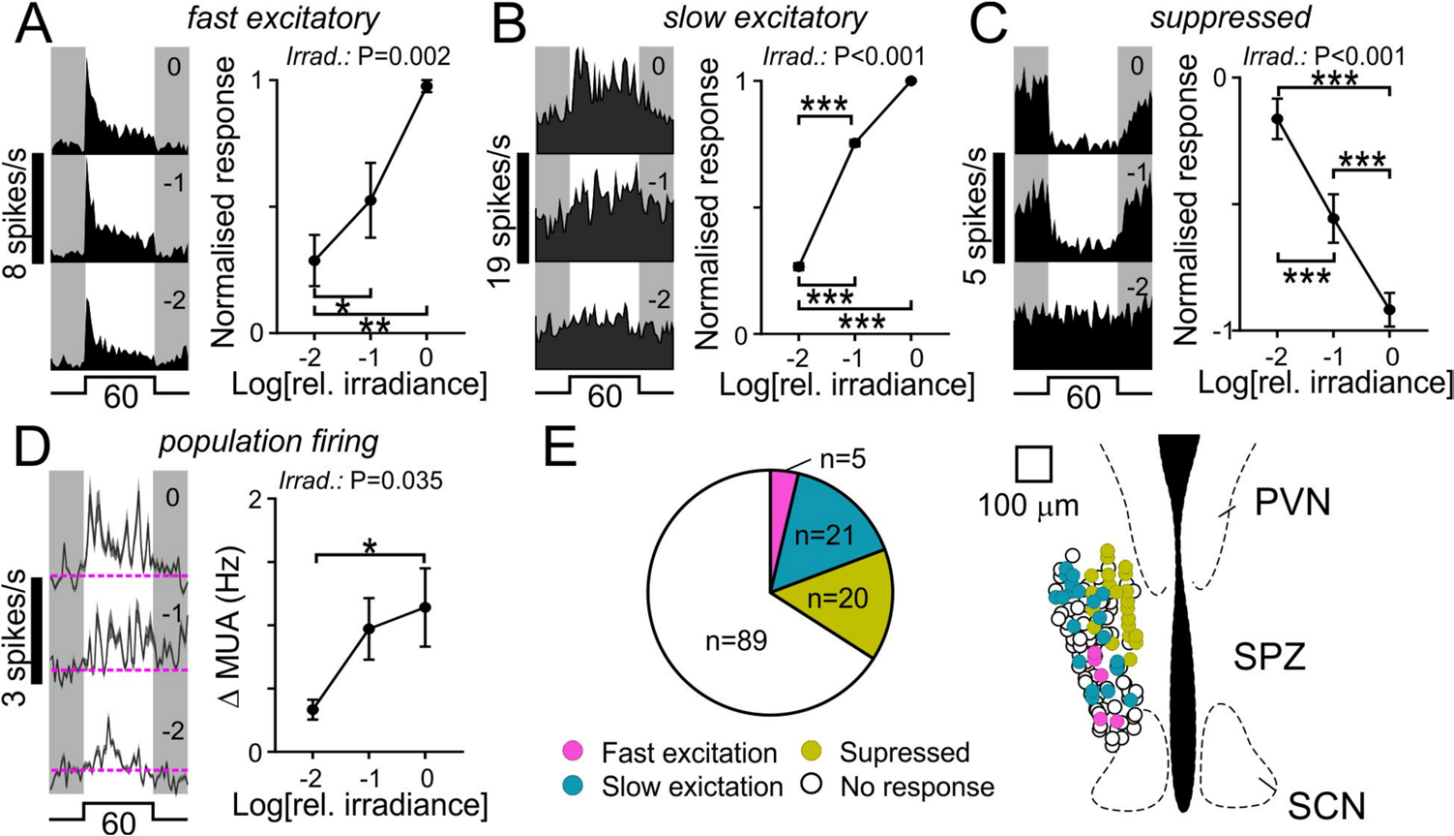
Irradiance-dependent regulation of subparaventricular zone neural activity.** A**–**C** Representative single neuron responses (left) and mean ± SEM normalised population responses (right) to 60 s light steps from darkness for cells classified as fast excitatory (**A**; *n* = 5), slow excitatory (**B**; *n* = 21) or light suppressed (**C**; *n* = 20). See Additional file 2 for underlying raw data. **D** Mean ± SEM multiunit activity (MUA) traces (left) and light-evoked change in firing (right) across supraventricular zone (SPZ) recording sites (*n* = 128) for 60 s light steps from darkness. **E** Proportion of isolated single units exhibiting specified light response types (left) and projected anatomical locations of the corresponding cells (right). Data in **A**–**D** analysed by one-way RM ANOVA with Tukey’s post tests (**A**: *F*_2, 12_ = 11.2, *P* = 0.002; **B**: *F*_2, 60_ = 1190, *P* < 0.0001; **C**: *F*_2, 57_ = 21.3, *P* < 0.0001; **D**: *F*_2, 381_ = 3.4, *P* = 0.035)

We started by identifying cells that exhibited light-dependent changes in firing, by applying strongly L-opsin-biased light steps (60 s, from darkness) across a range of light intensities associated with robust light avoidance behaviour and activity suppression (Additional file 1: Fig. S5A). From multielectrode recordings in four *Opn1mw*^*R*^ mice, we identified *n* = 46 SPZ neurons that exhibited reliable, irradiance-dependent, changes in firing (from *n* = 135 total cells). Among this population of light-influenced neurons, we were further able to identify 3 distinct classes of neural response (Fig. 5A, B, C, E). A small subset of cells (*n* = 5) exhibited rapid light-dependent increases in firing that decayed to a lower steady-state level of sustained firing (Fig. 5A; Additional file 1: Fig. S5B). Such responses were strongly reminiscent of those we have previously reported for neurons in the SCN and other primary visual targets [45, 48, 49], suggesting such cells reflect those receiving direct retinal input. We also, however, found a larger population of cells (*n* = 21) that exhibited very sluggish, sustained, irradiance-dependent increases in firing (Fig. 5B, Additional file 1: Fig. S5C) and another substantial population (*n* = 20) that exhibited pronounced irradiance-dependent decreases in firing (Fig. 5C, Additional file 1: Fig. S5D), likely reflecting cells receiving input from other retinorecipient regions [49]. In either case, collectively, these data indicate that light stimuli associated with robust activity suppression and light avoidance produce substantial changes in the activity of various subpopulations of SPZ neurons. Of note, however, analysis of overall SPZ population firing (multiunit activity; MUA, *n* = 128 recording sites from 4 mice; Fig. 5D) revealed a net irradiance-dependent increase in SPZ activity (consistent with our previous result that stimuli producing a greater negative masking result in elevated SPZ c-Fos expression).

**Fig. 6:**
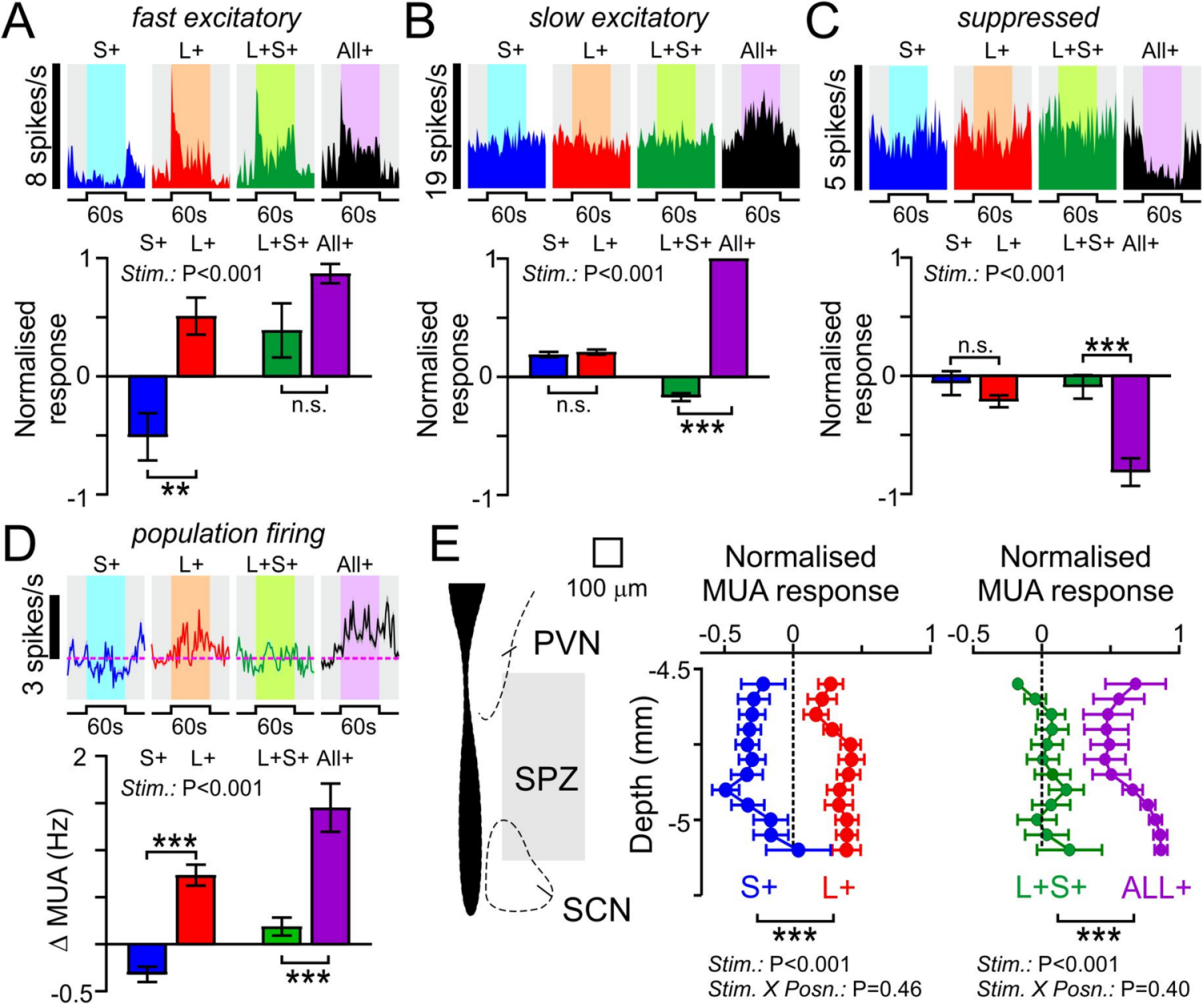
Cone-opponent regulation of subparaventricular zone network activity.** A**–**C** Representative single neuron responses (top; same cells as Fig. 5A–C) and mean ± SEM normalised population responses (bottom; *n* = 5, 21 and 20 respectively in **A**–**C**) for the identified classes of SPZ neurons in response to 60 s, 1 log unit, light steps selectively targeting one or both cone opsin types (S + , L + and L + S +) or all opsins equally (All +). See Additional file 2 for underlying raw data. **D** Mean ± SEM multiunit activity (MUA) traces (top) and stimulus-evoked changes in firing (bottom) across supraventricular zone (SPZ) recording sites (n = 128) for the same stimuli in **A**–**C**. **E** Mean ± SEM normalised MUA response to light steps targeting S- or L-cone opsins (left) or for both cone opsins with or without changes in brightness for melanopsin and rods (right) as a function of recording site depth within the SPZ (*n* = 8–26 sites/bin). Data in **A**-**D** analysed by one-way RM ANOVA with Tukey’s post tests (**A**: *F*_3, 12_ = 12.4, *P* = 0.0006; **B**: *F*_3, 60_ = 525.7, *P* < 0.0001; **C**: *F*_3, 57_ = 15.4, *P* < 0.0001; **D**: *F*_3, 381_ = 29.4, *P* < 0.0001). Data in **E** analysed by 2-way mixed effects ANOVA (left- Stim.: *F*_1,240_ = 216.3, *P* < 0.0001; position—*F*_11,240_ = 0.84, *P* = 0.596; Stim. X position—*F*_11,240_ = 0.99, *P* = 0.459; right—Stim.: *F*_1,240_ = 118, *P* < 0.0001; position-*F*_11, 240_ = 0.803, *P* = 0.637; Stim. X position-*F*_11,240_ = 1.05, *P* = 0.403). *,** and *** indicate *P* < 0.05, *P* < 0.01 and *P* < 0001, respectively

To more directly evaluate the influence of cone-photoreceptive signals on SPZ network activity we next provided a steady background light stimulus (equivalent to the dimmest stimulus applied in Fig. 5A–D) and assessed the response following 60 s steps to various alternate spectra (Additional file 1: Fig. S6), designed to provide selective 1 log unit increase in brightness for just S- or L-cone opsin (‘S + ’ and ‘L + ’, respectively), both cone opsins (‘L + S + ’) or all photoreceptor classes (‘All + ’). Under these conditions, for the population of cells exhibiting fast excitatory responses, selective increases in irradiance for S- and L-cone opsin resulted in opposing changes in firing, with reduced activity for the S + and robust increases in activity for the L + stimulus, providing a potential origin for the spectrally-opponent regulation of behaviour observed previously (Fig. 6A). To determine whether such cells could also provide a route for melanopsin-dependent influences we next compared responses to the All + and L + S + stimuli (which provide the same step in irradiance for cones but do or do not also provide contrast for melanopsin and rods). Despite a nominally greater increase in firing for the All + stimulus, we did not detect a significant difference from the response of these cells to the L + S + stimulus (Fig. 6A), suggesting a minimal contribution of melanopsin/rods to their responses under our experimental conditions. By contrast, for cells with slow excitatory or light-suppressed type responses, stimuli targeting just cones evoked negligible changes in firing (with no evidence of opponency), whereas the All + stimulus-evoked very robust increases or decreases in firing respectively (Fig. 6B, C), suggesting strongly melanopsin-dominated responses (given the background light intensity well within the photopic range; Additional file 1: Fig. S6). That conclusion is consistent with the low sensitivity of such cells to light steps from darkness (Fig. 5) and our previous analysis of the spectral sensitivity of equivalent light responses in the anterior hypothalamus of Opn1mw^R^ mice which was incompatible with any substantive rod contributions [49].

Given the diverse sensory properties of the different classes of light-influenced SPZ neurons revealed above, we again turned to the analysis of MUA data to determine the net impact of the different photoreceptive signals on SPZ network activity. In line with our data for fast excitatory cells (Fig. 6A), this revealed a clear and robust cone-opponent regulation with a net increase in firing for L + and a decrease in firing for S + stimuli (Fig. 6D). Moreover, while the L + S + stimulus-evoked negligible overall changes in MUA, the All + stimulus evoked a robust net increase in firing (Fig. 6D), consistent with a net excitatory influence of melanopsin. Since a detailed analysis of the anatomical localisation of the different classes of light-responsive neurons suggested some partial segregation with different subregions of the SPZ (Fig. 5E), we also asked whether these net changes in SPZ network activity varied across this part of the hypothalamus. In fact, however, analysis of MUA data as a function of electrode site depth revealed robust cone-opponent modulation and irradiance-driven activation across the dorsal–ventral extent of the SPZ (Fig. 6E). Collectively, then, our electrophysiological data reveal a combination of irradiance-dependent increases in activity and cone-dependent chromatic (L-ON/S-OFF) modulation of SPZ firing activity that aligns with the sensory characteristics revealed by our behavioural studies and neuroanatomical data implicating the SPZ activation in light-induced suppression of activity and light-avoidance.

## Discussion

Our data provide important new insight into the sensory mechanisms controlling key, ethologically relevant, aspects of the acute effects of light on mouse behaviour. While prior studies have strongly implicated ipRGCs in regulating light-avoidance behaviour and negative masking, the relative contributions of melanopsin and outer retinal photoreceptors, especially cones, to such behaviours in adult animals has remained poorly understood [6–8, 18–20, 22, 23, 25, 26]. Our data now show that cone-derived short vs. long wavelength opponent signals (analogous to the ‘blue’- ‘yellow’ axis of human colour vision) impose a substantial modulatory influence over melanopsin- (and potentially also rod-) driven activity suppression and light avoidance. As a consequence, this mechanism acts to promote mouse exploratory behaviour in the presence of environmental illumination enriched for shorter wavelengths, such as occurs during natural twilight.

We didn’t here seek to explicitly assess the potential that rod photoreceptors might contribute, alongside melanopsin, to irradiance-related effects of acute light exposure on behaviour. Nonetheless, our interpretation that the cone-independent effects observed here primarily reflect melanopsin-driven responses is strongly supported both by prior studies of light avoidance/masking and the known sensory properties of rods and melanopsin. Hence while rods can still contribute to physiological and behavioural responses to light–dark transitions at high irradiances (e.g. [13, 57]), we find irradiance–dependent (and cone-independent) reductions in behavioural activity for stimuli more than 10 times brighter than the saturation point for rod responses to light–dark transitions (and well-within the range of melanopsin-driven responses; [30, 33–35, 44–49]). Consistent with our interpretation that such effects originate with melanopsin, it is well-established than knockout or knockdown of melanopsin substantially impairs negative masking [18, 19, 43] while melanopsin only (*rd/rd cl*, *rd/rd*) animals robustly retain such responses with no loss of sensitivity relative to wildtype [20, 21, 26]. Similarly, in the case of our data on light avoidance, in principle some rod-based contrast responses could survive under our experimental conditions (e.g. [58]), however, the irradiance-dependent effects we observe span intensities where rods can no longer support robust visually guided behaviours [36–38]. Indeed, our observation that these irradiance-dependent effects are lost in animals lacking melanopsin aligns well with previous data suggesting a role for melanopsin/ipRGCs in such responses [8, 9, 25], while the modest residual light avoidance in both these animals and those lacking cone phototransduction argues against any major role for rods under our experimental conditions.

With respect to our data showing the effects of cones, an important consideration in interpreting those findings is the extent to which the stimuli provided might have inadvertently resulted in changes in irradiance for melanopsin (or rods). Our data showing the behavioural responses to these cone-directed (but not untargeted) changes in irradiance are absent in mice lacking cone phototransduction provides confidence that this is not the case. This is also consistent with our previous experience of using equivalent approaches where we have validated that behavioural and electrophysiological responses to such stimuli require functional cones [15, 16, 30] as well as validating approaches for manipulating melanopsin excitation independent of detectable responses from cones (or rods) [28–30, 46, 59, 60]. We should note here, however, that our finding that S-cone signals oppose light-induced suppression of activity are seemingly at odds with an earlier study that suggested S-cones can drive ‘negative masking’ responses [20].

There are several methodological differences between that earlier study and our own work that could underlie this apparent discrepancy. For example [20], assessed suppression of wheel running to light pulses at a fixed circadian time, whereas we compared reductions in general (PIR-measured) activity under an ultradian cycle. It is formally possible, therefore, that cone-opponent signals might differentially modulate general activity vs. voluntary wheel running. Further, [20] assessed sensitivity to monochromatic light of varying wavelength, whereas we compared responses to stimuli matched to selectively differ in brightness for specific opsin classes. The former approach is likely not best suited to reveal the type of chromatic effects we report here and, in this case, those data are especially challenging to interpret owing to the tools available to the researchers at the time, which leave the precise nature of the stimuli used uncertain. Hence, in [20], stimuli were quantified based on readings from an optical power meter and peak transmission values of filters used (as reported by manufacturers). This may have substantially skewed the interpretation of the data showing high sensitivity to ultraviolet light. Indeed, the approach used to generate key medium wavelength comparator stimuli (fluorescent lights with broadband ‘green’ filters) is expected to have biased those stimuli to longer wavelengths than intended and therefore substantially underestimated medium wavelength sensitivity. The extent to which data from [20] do indeed indicate that S-cones can drive masking responses is therefore hard to evaluate. Certainly, under the experimental conditions studied here, we find no evidence for this. Rather our data clearly demonstrate an antagonistic effect of S-cone signals on light–induced suppression of behavioural activity.

The present data also align with established views positing the SPZ as a key site for acute-light-dependent control of mouse behaviour [4, 5, 50, 51], confirming the presence of robust cone-opponent modulation of SPZ neuronal activity that matches the observed chromatic modulation of mouse exploratory activity. We should note, however, that there is still some uncertainty as to the primary retinorecipient site that actually forms the origin of light-induced suppression of activity in nocturnal rodents.

The SPZ has been suggested as a potential primary locus for such effects based on data showing SCN-lesioned animals can retain negative masking and light avoidance behaviour [50] while lesions that damage retinal input to the SPZ but spare some input to the SCN show disrupted masking [51]. Such data could imply that retinal inputs to the SPZ are the primary origin of light-induced activity suppression. In this regard, it is notable that we find a population of cells in the SPZ whose characteristics are consistent with those of directly retinorecipient neurons and display evidence of the appropriate cone-opponent control to provide an origin for the behavioural effects observed here. Nonetheless, it is important to also note that the SPZ receives major inputs from other retinorecipient structures (the SCN and IGL) [41, 42]. Moreover, both the SCN and IGL contain neurons that display cone-opponent regulation (including neurons with L-ON/S-OFF or S-ON/L-OFF properties) [16, 31]. In the case of the IGL, it seems unlikely that this structure is the principle driver of light-induced activity suppression (since IGL lesions increase masking; [55]). It is certainly possible, however, that cone-opponent IGL or SCN cells contribute to the behavioural effects of spectral content observed in the present study. In either case, while we cannot definitively distinguish the relative roles of direct vs. indirect retinal inputs to the SPZ, the presence of an overall decreased neuronal activation of the SPZ for ‘bluer’ stimuli is fully consistent with the prevailing view [4, 5] that the SPZ is a key node within the brain networks underlying acute effects of light on mouse behaviour (including the chromatic effects observed here).

In closing, it is important to note that the suppressive effects of S-cone-enriched stimuli on light avoidance and acute suppression of activity observed here strongly align with an equivalent chromatic modulation of the effects of light on SCN-dependent circadian control. Hence we previously showed that ‘bluer’, twilight-like, lighting evokes weaker circadian responses than the lighting of equivalent melanopic irradiance whose spectral content better recreates day [15, 16]. The acute cone-opponent effects revealed in the present study therefore constitute an influential, previously unrecognised, sensory control mechanism that likely acts in concert with the longer-term circadian modulation to precisely tune the timing of mouse behaviour in the natural world around twilight.

## Conclusions

Given that all common indoor lighting is strongly biased towards longer wavelengths (and therefore appears very ‘yellow’ to mice and other rodents; Additional file 1: Fig. S2F,G), the present findings as to the impact of spectral content on mouse behaviour have significant implications that should be considered in the design of future laboratory behavioural experiments and husbandry contexts. Hence, an exciting prospect for the future is that using lighting more enriched in shorter wavelengths might enhance throughput for experimental paradigms that rely on mouse behavioural activity and/or improve welfare by reducing aversive behaviours. An exciting additional prospect is to explore the possibility that analogous effects of ‘colour’ might also apply to humans (e.g. on arousal or mood). Given the diametrically opposing acute effects of light on the activity of nocturnal and diurnal mammals it seems unlikely that our findings in mice would directly translate, rather one might imagine the opposite effects would be more likely (e.g. an inhibitory effect of S-cone signals on arousal). To our knowledge, there have not yet been many appropriately controlled studies to test such a possibility although, in keeping with the suggestion above, one recent study using lighting that selectively differed in brightness for S-cones, does provide some indication of a modest increase in reaction times under S-cone ‘bright’ conditions [61]. It will therefore be interesting to see if future studies can confirm whether such an effect is repeatable and/or extends to other aspects of cognition, mood or performance.

## Methods

### Animals

All experiments were conducted in accordance with the Animals (Scientific Procedures) Act of 1986 (United Kingdom). Mice were bred and housed at the University of Manchester in a 12:12 h light:dark (LD) cycle at 22 °C with food and water available ad libitum. Throughout, experiments employed adult, male and female, mice (90–210 days old) of the following strains: human red cone knockin (*Opn1mw*^*R*^; [27]), human red cone knockin/melanopsin knockout (*Opn1mw*^*R*^; *Opn4*^*−/−*^; [29]) and cone-specific cyclic nucleotide-gated channel α-subunit knockout (*Cnga3*^*−/−*^; [62]). For all experimental procedures, animals were housed in light-tight cabinets where illumination could be carefully controlled and maintained under a strict 12:12 LD cycle for at least 2 weeks prior to experimental procedures. Animal numbers for each experimental procedure/genotype are indicated in the relevant ‘Light stimuli and test paradigm’ sections below.

### Light–dark preference tests

#### Apparatus and general experimental procedures

The testing enclosure was constructed of black acrylic and comprised two 30 × 30 × 30 cm chambers with white polytetrafluoroethylene (PTFE) floor and ceiling (Plastics Direct Ltd., Oldham, UK). RGB and 385 nm LED strips (Downlights Direct Ltd., Oldham, UK) were mounted above the PTFE ceiling of each chamber in 4 parallel rows of 20 LEDs/strip to provide diffuse illumination. LED intensity for each of the four wavelength channels was controlled (independently for each chamber) via pulse width modulation to allow for controlled variations in the intensity and spectral composition of the available illumination. A 6 cm diameter doorway positioned centrally between the two chambers at the front of the enclosure allowed the mouse to enter and move freely between the chambers (Fig. 1A). On entering the enclosure, a sliding door covered the entrance to prevent the mouse from leaving until the experimental session terminated. LED intensities were set before introducing the mouse to the experimental enclosure and remained constant throughout the 15-min experimental sessions. During this time, video recordings of the mouse’s position in the chamber were captured synchronously an 8 frames/s by a pair of Raspberry Pi3B + with Pi NoIR camera modules and IR ring lights (RS components, Corby, UK) positioned centrally above each chamber. Unless otherwise specified, mice were removed from their home cage for testing between Zeitgeber time (ZT) 4.5 and 7.5 (where ZT 0 = time of lights on). The chamber was thoroughly cleaned with ethanol after the end of each experimental session, prior to testing the next experimental animal. Mice experienced a single test stimulus on any given day and subsequent testing was separated by at least 48 h. Individual animals completed one or two of the four total studies conducted (and therefore experienced up to 12 of the test stimuli) described below. In all cases, the brighter side of the chamber was randomised across trials and, within studies, the order of stimulus presentation was randomised between mice.

#### Light stimuli and test paradigms

All light measurements were performed using a calibrated spectroradiometer (Bentham instruments, Reading, UK) and quantified according to the known opsin sensitivities after correction for prereceptoral filtering [63, 64] as described previously [16, 46] (see Additional file 2 for raw spectral data). Reported intensities reflect the effective photon flux captured by a cosine diffuser directed at the ceiling of the experimental chamber. Stimuli for initial experiments (Studies 1 and 2) were designed to recreate the relative pattern of photoreceptor activation consistent with a wildtype mouse’s experience of an overcast day [16], across a 100-fold range of intensities (Fig. 1B). Stimuli for subsequent experiments examining cone influences (Studies 3 and 4) were based around an equivalent background (0.3 log units dimmer than the maximum in Studies 1 and 2) with spectral modulations designed to enable comparisons between melanopsin/rod matched stimuli with up to 1 log unit differences in irradiance for L and/or S-cone opsin (Fig. 2A).

Study 1 assessed the preference of *Opn1mw*^*R*^ mice (n = 6) as a function of time of testing, under conditions where there was a 100-fold difference in brightness between the two sides of the chamber. Specifically, mice were tested twice each in a 3 h window centred on their homecage light–dark transitions (i.e. ZT 0 and ZT 12) or at midday or midnight (ZT6 and ZT18). Within each epoch, the time of testing within the first or last 1.5 h was randomised across animals. For presentation (Fig. 1C), responses for each animal were averaged across the two midday and midnight timepoints. Study 2 assessed preference to dim light relative to a condition of equivalent spectral composition but higher (0.5, 1, 1.5 or 2 log unit) irradiance (Fig. 1D-F; n = 16 *Opn1mw*^*R*^; *n* = 13 *Opn1mw*^*R*^; *Opn4*^*−/−*^; n = 12 *Cnga3*^*−/−*^). These sample sizes are sufficient to allow the detection of modest within-group effect sizes with high reliability (*f* = 0.5 at > 95% power; G*power 3.1.9.2, [65]).

Study 3 assessed preference to lighting conditions that selectively differed in brightness by 1 log unit for L and/or S-opsin irradiance (Fig. 2 B, C; *n* = 7 *Opn1mw*^*R*^; *n* = 13 *Cnga3*^*−/−*^). In the absence of prior insight into the likely effect magnitude for these experiments, the sample size for *Opn1mw*^*R*^ was chosen to provide > 80% power to detect a moderate within-subjects effect size of *f* = 0.5. Subsequent experiments in *Cnga3*^*−/−*^ provided 95% power to detect an equivalent effect size.

Study 4 assessed how preference scaled with a selective difference in irradiance (− 0.7 to + 1 log unit) for L-opsin or S-opsin as well as how the difference in S-cone irradiance impacted preference when faced with a 1 log-unit difference in brightness for L-opsin (Fig. 2D, E; *n* = 9 *Opn1mw*^*R*^). This sample size provided ~ 95% power to detect an effect of equivalent magnitude to that observed in Study 2. Data from 3 of the total of 49 mice tested (2 *Opn1mw*^*R*^; *Opn4*^*−/−*^ contributing to Study 2 and one *Opn1mw*^*R*^ contributing to Study 4) were excluded from analysis due to a strong a consistent bias towards one side of the chamber irrespective of the nature of stimulus presentation.

#### Data analysis

Mouse position within the experimental apparatus was determined via custom Matlab (Mathworks, MA, USA) scripts, which determined the local region of maximal difference between each frame of the paired video recordings and a reference image of the empty chamber prior to the start of the experiment. From this we calculated the amount of time mice spent on the left or right sides of the chamber and, by dividing the chamber floors into an equally spaced 3 × 3 grid, the proportion of that time spent in the corners of the chamber. For the presentation of data on mouse preference across pairs of test stimuli we calculated a preference index of the form: [*T*_Bright_ − *T*_Dim_]/[*T*_Bright_ + *T*_Dim_], which ranges from + 1 (maximal preference to ‘bright’) to − 1 (maximal preference to ‘dim’). Group data from each study were then analysed by One or Two-way repeated measures ANOVA in GraphPad Prism v7 (GraphPad software, CA, USA), with one sample t-tests vs. a null hypothesis of zero preference or Sidak’s post-tests as appropriate. Where relevant, we used extra sum-of-squares *F*-test to assess for the presence of diurnal rhythms (Study 1; comparison of fits between zero slope 1^st^ order polynomial and sinusoids constrained to a period of 24 h) or to relationships between irradiance difference and preference (Study 4; test for non-zeros slope of best fit 1^st^ order polynomials or comparisons of slope between conditions).

### Masking assay

#### Apparatus and general experimental procedures

Mice were housed in wire-top cages in a custom, light-tight behavioural cabinet as previously described [15]. The roof of the cabinet housed four light boxes, each consisting of two smart multicolour bulbs (LIFX A60; LIFX, Cremorne, Australia) and 6 violet bulbs (405 nm, Led Engin LZ1-00UA00-00U7; RS Components, UK). A PTFE diffuser was mounted to the floor of light boxes/roof of the cabinet and the interior of the cabinet was painted white to provide uniform illumination. The smart bulbs were controlled wirelessly over a local network and violet bulbs were connected to LED drivers (T-Cube; Thorlabs, Ely, UK) via a multichannel analogue output module (NI 9264; National Instruments, TX, USA). LED intensities were then controlled on a second-by-second basis using a PC running Python (2.7.10). Mice were housed under a standard 12 h:12 h LD cycle prior to the start of the experiment where they were exposed to 16 days of a 1.5 h:1.5 h LD cycle whose light phase varied in spectral composition (see below). Throughout, mouse behavioural activity (counts/s) was assessed by a validated PIR monitoring system as described previously [66].

#### Light stimuli and test paradigm

A total of 12 *Opn1mw*^*R*^ and 12 *Cnga3*^*−/−*^ mice were employed in these experiments (comprising 2 cohorts of 6 mice for each genotype). These sample sizes allowed for the reliable detection of modest within-subjects effects in subsequent analyses (*f* = 0.5 at > 95% power). When the experiments commenced, for each repeat of the 1.5 h:1.5 h LD cycle mice experienced one of 8 different lighting conditions (measured and quantified as above). Stimuli included in these experiments were calibrated relative to a reference spectrum (‘spectra 1’) to either present spectrally neutral decreases or increases in intensity (− 0.8 or + 1 log unit) or to differ selectively in brightness for L and/or S-cones (see Additional file 1: Fig. S3 for full details). Throughout the 16-day experiment, the order of stimulus presentation was shuffled every 24 h such that each of the different conditions was evenly distributed across all the different possible circadian phases (see Fig. 3A, B).

#### Data analysis

Initial data processing was performed in Matlab. For the presentation of actograms, PIR-determined activity counts were collapsed into 10 min bins. We then averaged counts across all bins falling during epochs where the relevant test stimuli (or darkness) were presented. Average activity levels under each condition were then analysed either in raw form, to compare darkness vs. varying irradiance (Fig. 3C, G), or expressed as a percentage of activity occurring under darkness to compare the degree of masking (Fig. 3D, H). The resulting data were then analysed by one-way repeated measures ANOVA (GraphPad Prism) with, respectively, Tukey’s or Sidak’s post-tests (vs activity suppression under the reference spectra). For the presentation of time course data (Fig. 3E, I) activity (counts/10 min) was normalised according to the maxima across the experiment for each mouse and then averaged across presentations of the relevant test stimuli. For analysis of circadian rhythmicity, data were analysed by χ^2^ peridogram [67] of data across the full 16-day experiment. Dominant circadian period was determined as the midpoint of the peak in the periodogram values (range 20–28 h, bin size = 1 min) exceeding the *P* < 0.05 confidence threshold.

### Neuroanatomical mapping

#### Light stimuli and test paradigm

Mice (*n* = 12 *Opn1mw*^*R*^) were housed under a standard 12 h:12 h LD cycle for 2 weeks prior to the start of experiments. On the day before the experiment mice were transferred to constant darkness and then received a 30-min light pulse between projected ZT17.5 and 18 on the following day (i.e. 29.5 h after the end of the last light phase). Animals were randomly assigned to experience either the S-cone or L-cone enriched, rod and melanopsin-isoluminant, stimuli used for masking studies (Additional file 1: Fig. S3A, spectra 3 and 4 respectively; *n* = 6 mice/group). These group sizes allowed for the detection of large between-subjects effects (*d* = 1.8 at > 80% power) of the type expected based on the magnitude of the behavioural responses to these stimuli and previous related experiments [39]. Following the light pulse, mice were deeply anaesthetized with i.p. injection of urethane (2.2 g/kg; 30%w/v; Sigma-Aldrich, UK) under dim, deep red light (50 nW/cm^2^, *λ* > 650 nm) and then perfused transcardially with 0.9% saline followed by 4% paraformaldehyde. Brains were removed, postfixed overnight in 4% paraformaldehyde, and then transferred to 30% sucrose for cryoprotection before subsequent processing.

#### Immunohistochemistry

Brains were sliced at 40 µm using a freezing stage microtome (800 Sledge; Bright Instruments, UK). Slices were then washed with 1% triton diluted in phosphate-buffered saline (1% PBS) and blocked with 10% normal goat serum for 2 h at room temperature. Slices were then labelled using polyclonal rabbit anti-c-Fos antibody (AB190289 Abcam; RRID:AB_2737414; 1:500 dilution) and left to incubate at 4 °C overnight. The following day, slices were washed in 0.2% PBS and incubated with a goat anti-rabbit fluorescent secondary antibody (A-11008 Invitrogen, RRID:AB_143165; 1:500 dilution) for 3 h at room temperature, then washed in PBS and mounted. Primary and secondary antibodies were added in a mixture of 0.2% PBS with 10% normal goat serum.

#### Quantification and analysis

Images of c-Fos immunolabelled brain sections were acquired on a 3D-Histech Pannoramic-250 microscope slide-scanner using a 20 × /0.80 Plan Apochromat objective (Zeiss) and the FITC filter. Sections containing the anterior hypothalamic regions surrounding the RHT (corresponding mouse brain atlas sections between -0.22 and -1.46 mm caudal to bregma; [68]) were selected and c-Fos expressing nuclei manually counted within a window spanning 2.1 mm on either side of the third ventricle and 2.1 mm dorsally from the base of the brain (9–12 sections/mouse). The annotated sections were then imported into Matlab with a custom script that compiled a spatial map of c-Fos expression density. In brief, individual sections were aligned by a manually positioned reference marker (a cross bisecting the midline and base of the hypothalamus). Positions of annotated neurons were then converted to stereotaxic coordinates (using scale information embedded in the image) and binned onto a 50-µm × 50-µm grid to define local expression density (cells/mm^2^). For the visualisation of anatomical distributions, data for each mouse was averaged across left and right hemispheres and across adjacent sections corresponding to one of four standardised anatomical templates based on corresponding rostral-caudal position in the mouse brain atlas [68]. For quantification, the mean c-Fos expression density for each mouse was calculated as the average across all bins falling within the neuroanatomical boundaries of the relevant structure, as delineated in the best-matching mouse brain atlas section [68].

### In vivo electrophysiology

#### Surgical and recording procedures

Mice were taken from the colony room ~ 1 h before lights off and anesthetised using urethane (1.55 g/kg i.p.; Sigma-Aldrich, Dorset, UK). Mice were then placed on a stereotaxic frame; the skull was exposed, and a small craniotomy was performed 1.05 mm lateral and 0.5 mm posterior to bregma. Pupils were dilated by the application of 1% atropine (Sigma Aldrich) and mineral oil applied to retain corneal moisture. A multielectrode recording probe (A32-Poly3; Neuronexus, MI, USA; comprising 2 parallel rows of 10–12 electrode sites) was then coated in CM-DiI (V22888; Fisher Scientific, Loughborough, UK) for post hoc histological visualisation, inserted into the brain at 9° to the dorsal–ventral axis a lowered to a depth of 5.1 mm from the pial surface using a micromanipulator (MO-10, Narishige International Ltd., London, UK). Mice were then left to dark adapt 30 min before the start of recordings, such that recordings spanned the early part of the dark phase in the mouse’s home cage LD cycle (Zeitgeber time 13–18).

During the experiment, wideband neural signals were acquired using a Recorder64 system (Plexon, TX, USA), amplified to a gain of 3500 × , digitised at 40 kHz and stored continuously in a 16-bit format. MUA was simultaneously acquired online from all channels by collecting timestamped 1 ms waveform segments triggered when the highpass (300 Hz) filtered data streams crossed a threshold set at − 35 µV. Single unit activity was subsequently discriminated offline from the wideband data stream using an automated template-matching-based algorithm (Kilosort; [69]). Data for identified clusters were then exported to Offline Sorter (Plexon) as ‘virtual tetrodes’ (1 ms waveform segments detected across 4 adjacent channels) for manual refinement and validation as distinct single units on the basis of spike cross- and auto-correlograms.

#### Light stimuli and test paradigm

Stimuli were generated with three independently controlled LEDs (λmax 410, 470 and 617 nm; Thorlabs, NJ, USA), combined via dichroic mirrors to generate uniform illumination. LED intensity and timing was controlled via a PC running LabVIEW software and a USB-6343 DAQ board (National Instruments, TX, USA), with a neutral density (ND) filter wheel providing additional control over stimulus irradiance (up to a 100-fold; ND0-ND2). Experimental stimuli were administered to the contralateral eye via a 7 mm diameter, flexible fibre optic light guide, which was positioned 5 mm from the retinal surface and fitted with an internally reflective plastic cone to provide approximately full field monocular illumination.

Recordings were performed in *n* = 4 *Opn1mw*^*R*^ mice. This sample size was chosen based on previous experience with related experiments (e.g. [16, 30, 31, 49, 70]), and data from c-Fos mapping experiments (above) to result in sufficient numbers of isolated neurons to detect even modest within-group effects (*f* = 0.6 at > 80% power) across the stimulus protocols detailed below. Mice were initially presented with 60-s light steps from the darkness of an L-cone biased reference stimulus at three log-spaced irradiances (ND0-ND2; see Additional file 1: Fig. S5A). Stimuli were delivered as a repeating staircase for a total of 6 repeats/irradiance (interstimulus interval = 10 min). Subsequently, we applied a steady background stimulus (equivalent to the reference stimulus at ND2) and assessed the impact of 60 s transitions to each of four test stimuli (Additional file 1: Fig. S6) providing either selective 1 log increases in brightness for S- or L-cone opsin (S + , L +), both cone opsins (S + L +) or all mouse opsins (All +). Stimuli were delivered in interleaved fashion for a total of 10 repeats/stimulus (interstimulus interval = 2 min).

#### Data analysis

Isolated units were classified as light responsive based their averaged firing rates in the first 1 s and last 45 s following bright light steps, relative to the preceding 30 s of darkness. Cells showing significant (t-test, *P* < 0.05) increases or decreases in firing during the last 45 s of the light step were classed as light activated or supressed respectively. The former group was subdivided into ‘fast’ and ‘slow’ according to whether the cells also showed a significant increase in firing during the first 1 s of the light step. For all subsequent analyses for single cell population and MUA, we used the mean change in firing across the full 60-s light step (relative to the mean baseline firing rate in darkness or under the background as appropriate). For the presentation of data from populations of isolated neurons, we further normalised responses of each contributing cell to their maximal response across all relevant test stimuli. For analysis of anatomical variation in MUA responses data were binned according to the projected stereotaxic depth of the recordings sites, as determined below (100 µm bins). Statistical analysis of the resulting data employed one-way or two-way ANOVA (with repeated measures as appropriate) followed by Turkey’s post hoc tests (GraphPad Prism).

#### Histological processing

Immediately after the termination of the in vivo electrophysiology experiments, mice were culled by cervical dislocation and an encephalectomy was performed. Brains were then placed into 4% paraformaldehyde for 48 h and cryoprotected in 30% sucrose for at least 24 h. Brains were then frozen in dry ice and coronal slices (100 µm thickness), were taken with a freezing sledge microtome (800 Sledge; Bright Instruments, UK). Slices were then mounted to glass slides and covered with coverslips. Once dried, sections were observed and imaged using an upright light microscope to localise the Di-I labelled electrode tract in the hypothalamus (BX51, Olympus, UK). The resulting images were then aligned with corresponding sections form the mouse brain atlas [68] to define projected stereotaxic locations of recording sites and isolated neurons.

### Supplementary Information

**Additional file 1: Fig. S1.** Diurnal and light-dependent variation in mouse exploration. **Fig. S2.** Cone-opponent influences on mouse behaviour. **Fig. S3.** Characterises of test stimuli for defining cone influences on home cage activity. **Fig. S4**. c-Fos expression across hypothalamic nuclei following ‘Yellow’ and ‘Blue’ light steps. **Fig. S5.** Analysis of subparaventricular zone sensory responses. **Fig. S6.** Stimuli for analysing cone influences on subparaventricular neuronal activity.

**Additional file 2: Raw Data.** Raw data underlying Figs. 1-6 and Figs. S1-S6.

## Data Availability

All data generated or analysed during this study are included in this published article and its Additional files (see Additional file 2 for underlying raw data values).

## Abbreviations

IGL: Intergeniculate leaflet
ipRGCs: Intrinsically photosensitive retinal ganglion cells
LD: Light:dark
MUA: Multiunit activity
ND: Neutral density
NRe: Nucleus reuniens
PIR: Passive infrared
PBS: Phosphate-buffered saline
PFTE: Polytetrafluoroethylene
RHT: Retinohypothalamic tract
SCN: Suprachiasmatic nucleus
SPZ: Subparaventricular zone
ZT: Zeitgeber time
