# Supplementary material for: Regulation of mouse exploratory behaviour by irradiance and cone-opponent signals
Source: BMC Biol. 2023 Aug 21;21:178. doi: 10.1186/s12915-023-01663-6 (PMC10441731; doi:10.1186/s12915-023-01663-6)
Supplement: Supplementary file 1 — Additional file 1: Fig. S1. Diurnal and light-dependent variation in mouse exploration. Fig. S2. Cone-opponent influences on mouse behaviour. Fig. S3. Characterises of test stimuli for defining cone influences on home cage activity. Fig. S4. c-Fos expression across hypothalamic nuclei following ‘Yellow’ and ‘Blue’ light steps. Fig. S5. Analysis of subparaventricular zone sensory responses. Fig. S6. Stimuli for analysing cone influences on subparaventricular neuronal activity. [file 12915_2023_1663_MOESM1_ESM.docx]

**
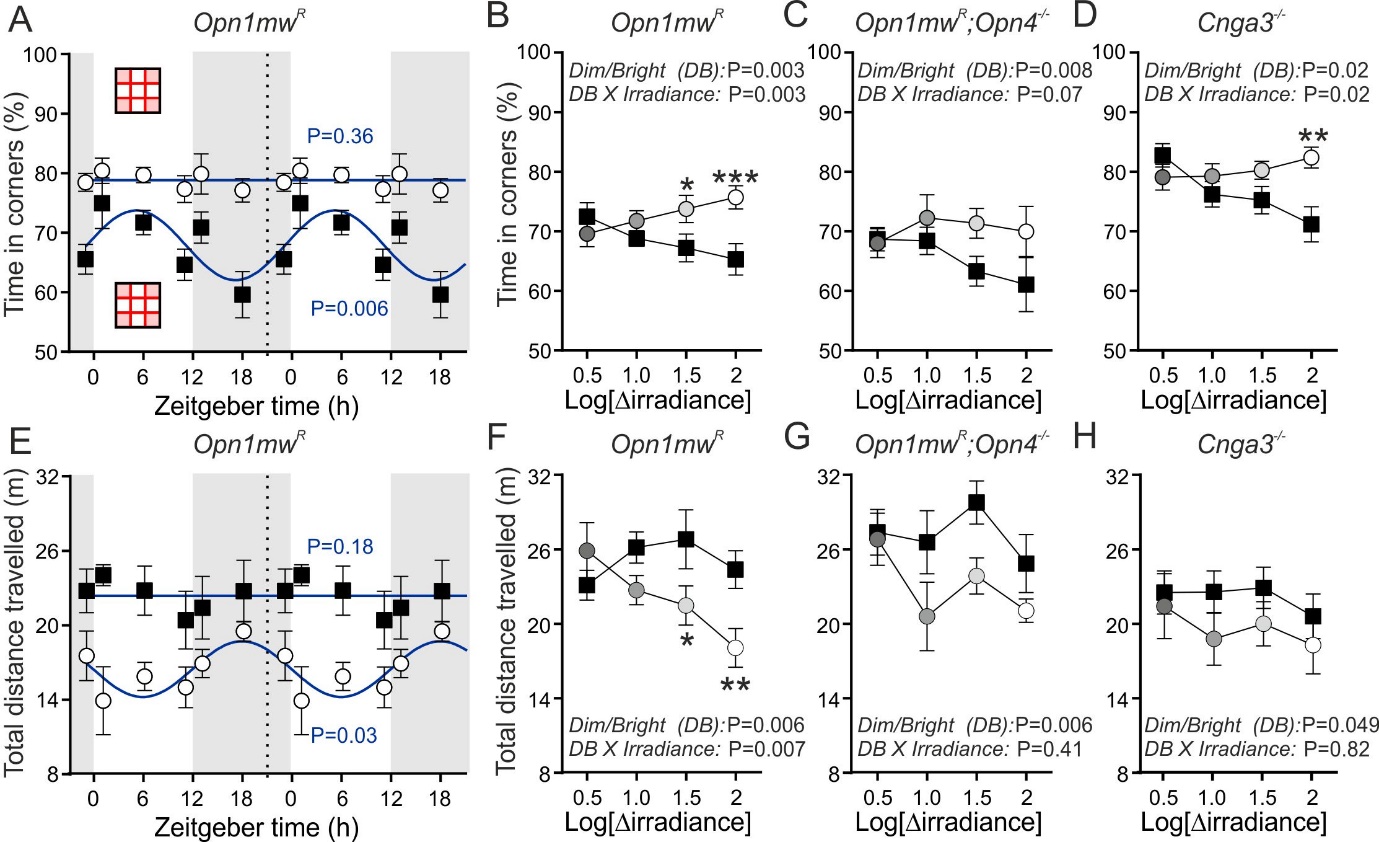
**

**Figure S1. Diurnal and light-dependent variation in mouse exploration.** (**A**) Mean±SEM proportion of time *Opn1mw^R^* mice (n=6) spent in the corners of the ‘bright’ (○) and ‘dim’ (□) sides of the test apparatus as a function of time of testing (double plotted). (**B**-**D**) Mean±SEM proportion of time *Opn1mw^R^* (**B**; n=16), *Opn1mw^R^;Opn4^-/-^* (**C**; n=11) and *Cnga3^-/-^* mice (**D**, n=12) spent on corners of ‘bright’ (○) and ‘dim’ (□) sides of the chamber (testing at ZT 4.5-7.5). (**E-H**) Mean±SEM total distance travelled by mice in ‘bright’ (○) and ‘dim’ (□) sides of the test apparatus (same animals and test conditions as **A-D** respectively). Data in **A** and **E** analysed by comparison of Sinusoidal vs. 1^st^ order polynomial fit (F-test; **A**: ○ - F_1,34_=0.9, P=0.36; □ - F_1,34_=8.5, P=0.006; **E**: ○ - F_1,34_=4.98, P=0.03; □ - F_1,34_=1.94, P=0.18). Data in **B**-**D** and **F**-**G** analysed by 2-way RM-ANOVA with Sidak’s post tests (**B**; DB: F_1,15_=13.1, P=0.003, Irrad.: F_3,45_=0.1, P=0.98, DB X Irrad.: F_3,45_=5.4, P=0.003; **C**; DB: F_1,10_=11.2, P=0.008, Irrad.: F_3,30_=0.5, P=0.70, DB X Irrad.: F_3,45_=2.6, P=0.07; **D**; DB: F_1,11_=8.2, P=0.02, Irrad.: F_3,33_=1.9, P=0.16, DB X Irrad.: F_3,33_=3.9, P=0.02; **F**; DB: F_1,15_=10.0, P=0.006, Irrad.: F_3,45_=1.7, P=0.19, DB X Irrad.: F_3,45_=4.6, P=0.007; **G**; DB: F_1,10_=12.2, P=0.006, Irrad.: F_3,30_=3.2, P=0.04, DB X Irrad.: F_3,45_=1.0, P=0.41; **H**; DB: F_1,11_=4.9, P=0.049, Irrad.: F_3,33_=0.8, P=0.51, DB X Irrad.: F_3,33_=0.3, P=0.82). *,** and *** indicate P<0.05, P<0.01 and P<0001 respectively.

**
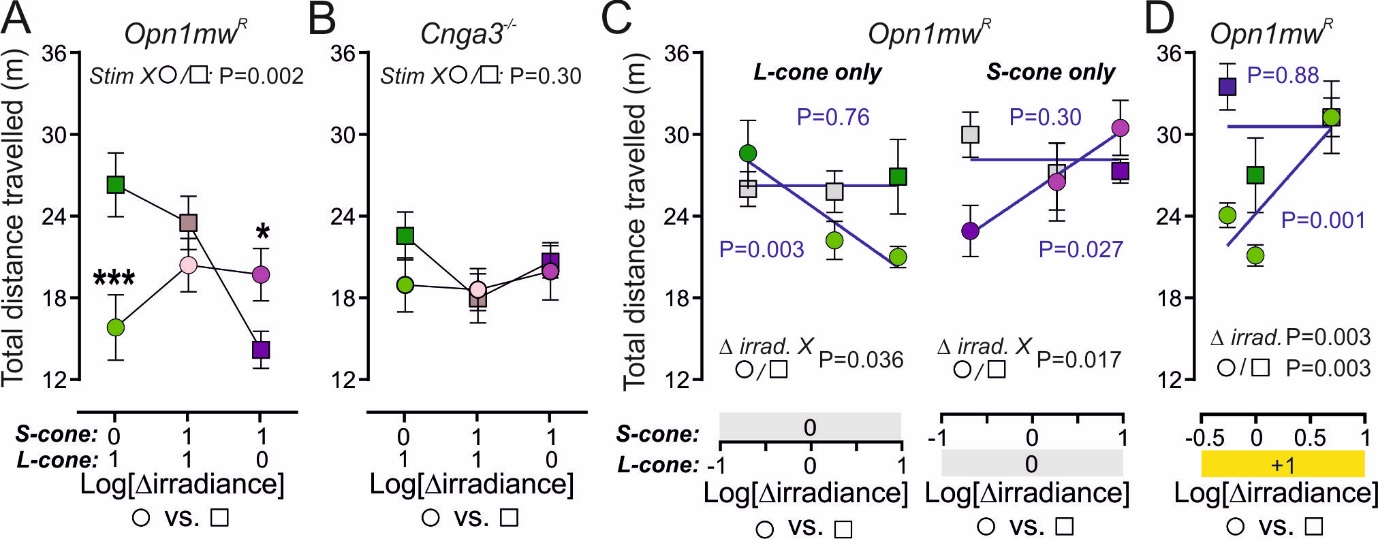
**

**Fig S2. Cone-opponent influences on mouse behaviour.** (**A,B**) Mean±SEM total distance travelled by *Opn1mw^R^* (**B**; n=7), and *Cnga3^-/-^* mice (**C**, n=13) under L- and/or S-cone ‘bright’ (○) vs. ‘dim’ (□) lighting conditions. Data analysed by 2-way RM ANOVA with Fisher’s post-tests (**A**: Stim: F_2,12_=3.1, P=0.08, ○/□: F_1,6_=1.3, P=0.30, Stim X ○/□: F_2,12_=11.4, P=0.002; **B**: Stim: F_2,24_=1.3, P=0.29, ○/□: F_1,12_=0.6, P=0.45, Stim X ○/□: F_2,24_=1.3, P=0.30). (**C**) Mean±SEM total distance travelled by *Opn1mw^R^* mice (n=8) as a function of irradiance difference between the two sides of the test apparatus (○ vs. □), where irradiance differed only for L- (left) or S-cone opsin (right). Data analysed by 2-way RM ANOVA (left: Δ irrad.: F_2,28_=2.9, P=0.07, ○/□: F_1,14_=1.6, P=0.22, Δ irrad. X ○/□: F_2,38_=3.8, P=0.036; right: Δ irrad.: F_2,28_=1.2, P=0.31, ○/□: F_1,14_=0.5, P=0.51, Δ irrad. X ○/□: F_2,38_=4.7, P=0.017). Linear fits tested for non-zero slope (Left: ○- F_1,22_=10.9,P=0.003; □ F_1,22_=0.09,P=0.76; Right: ○- F_1,22_=5.6,P=0.027; □ F_1,22_=1.1,P=0.30). (**D**) Mean±SEM total distance travelled by *Opn1mw^R^* mice (n=8) as a function of irradiance difference for S-cone opsin between the two sides of the test apparatus (○ vs. □), while irradiance was consistently 1 log unit higher for L-cone opsin. Data analysed by 2-way RM ANOVA (Δ irrad.: F_2,28_=7.0, P=0.003, ○/□: F_1,14_=13.4, P=0.003, Δ irrad. X ○/□: F_2,38_=3.0, P=0.066). Linear fits tested for non-zero slope (○- F_1,22_=22.4,P=0.001; □ F_1,22_=0.092P=0.88). *,** and *** indicate P<0.05, P<0.01 and P<0001 respectively.

**^
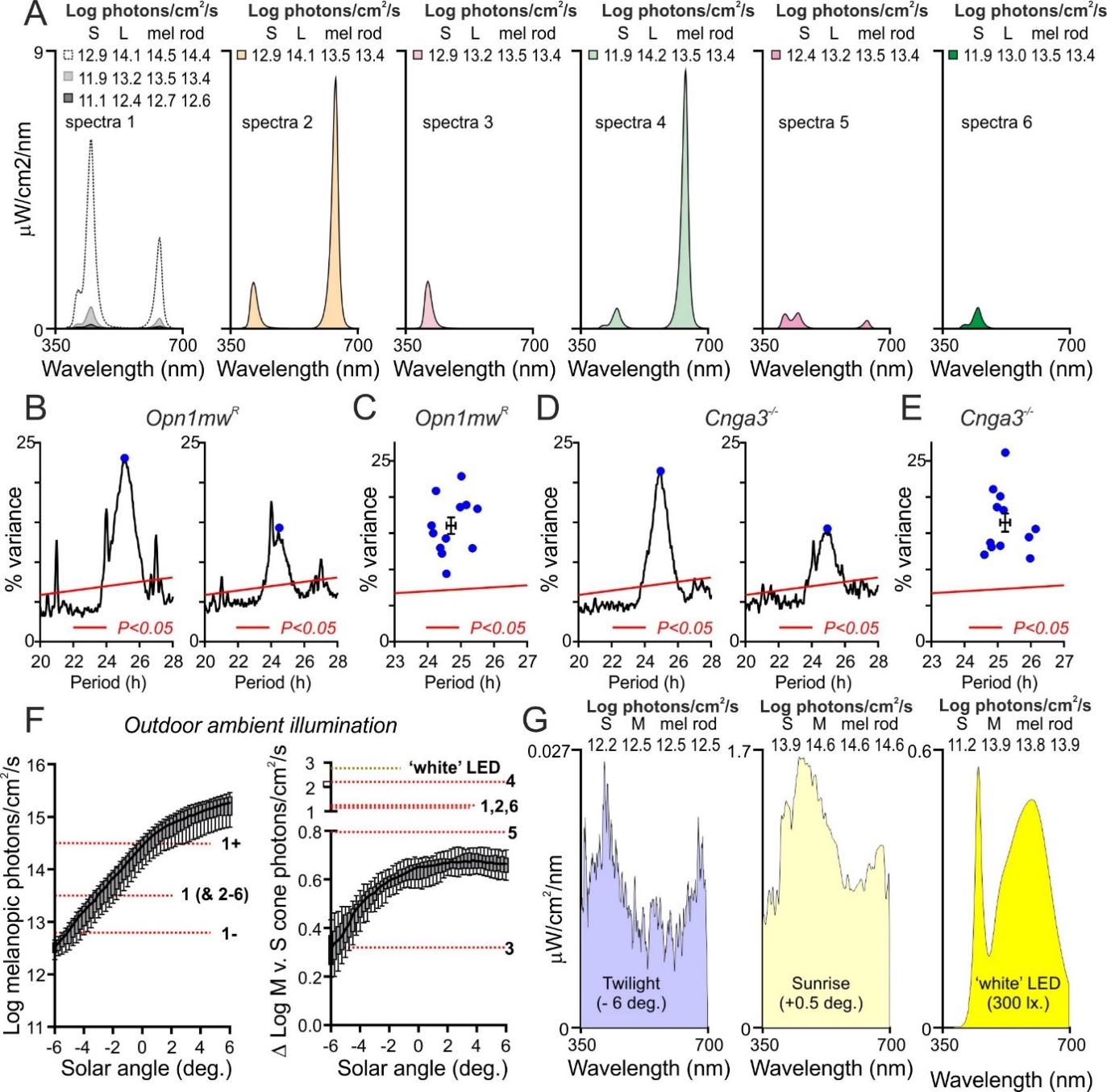
^Figure S3. Characterises of test stimuli for defining cone influences on home cage activity.** (**A**) Spectral power distributions and corresponding effective photon flux for *Opn1mw^R^* mouse opsins across the eight test stimuli from Fig. 3. (**B**,**D**) χ^2^-periodograms across the 16-day test epoch for representative *Opn1mw^R^* (**B**) and *Cnga3^-/-^* (**D**) animals shown in Fig 2B and F. Red line represents P<0.05 significance threshold, blue dots indicate dominant circadian peak. (**C**,**E**) Dominant circadian period and associated rhythm robustness (% variance) from χ^2^-periodogram analysis for *Opn1mw^R^* (**C;** n=12) and Cnga3-/- (**E**; n=12) animals contributing to Fig 2. Black error bars represent population means±SEM. (**F**) Box and whisker (10^th^-90^th^ percentile) plots showing relationship between solar angle and (left) log effective melanopic photons/cm^2^/s provided by natural daylight or (right) the log difference in M- vs. S-cone effective photons/cm^2^/s, as experienced by a wildtype mouse. Data derived dataset in [16] (71 dawn/dusk observations, Manchester UK, Aug.-Oct. 2005). Dashed red lines show the corresponding irradiance (left) or relative ‘colour’ (right) of test spectra in **A**. (**G**) Average twilight (solar angle = -6 deg.) and sunrise (solar angle=+0.5 deg.) spectra (from data presented in **F**) and spectra of a standard (4000K) white LED providing 300 lx for comparison (typical of that commonly used in rodent experiments and husbandry), with corresponding effective photon flux calculations for wildtype mouse opsins.


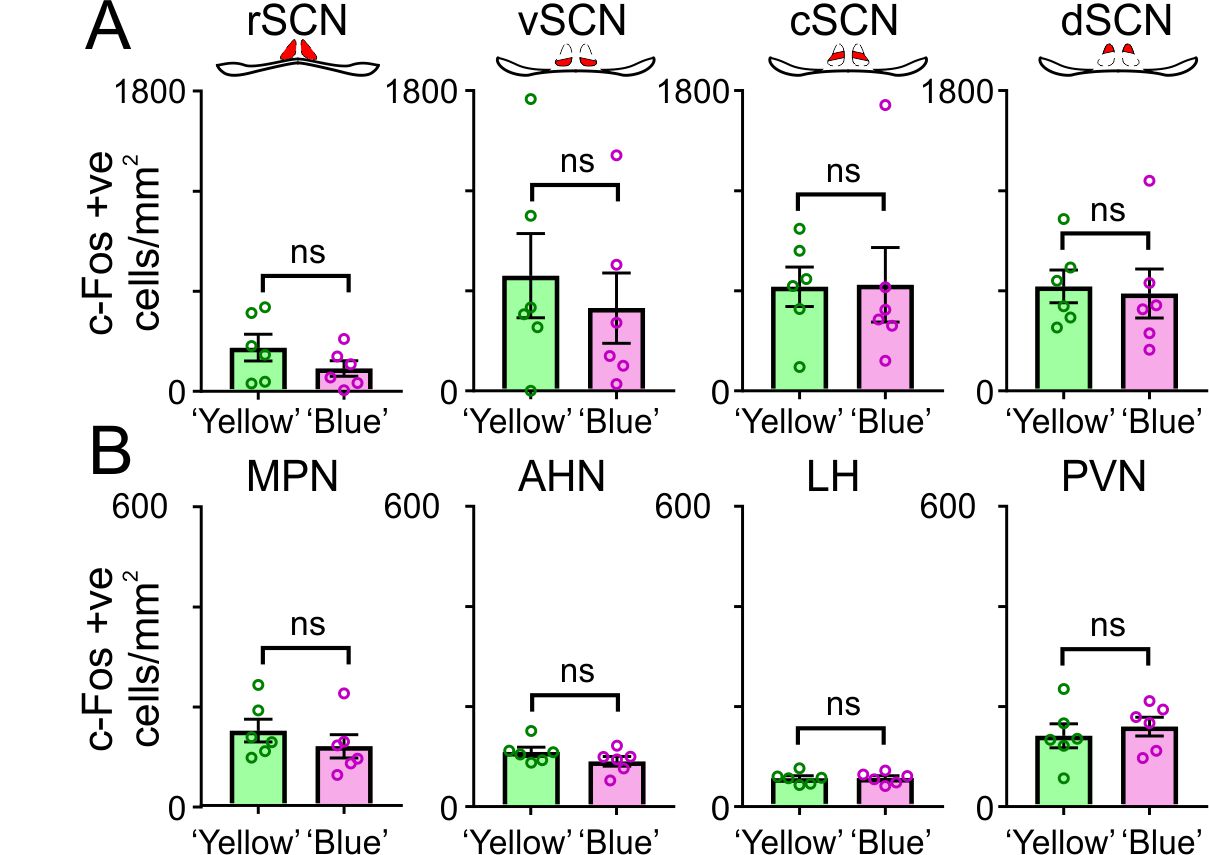


**Figure S4. Equivalent c-Fos expression across major hypothalamic nuclei following ‘Yellow’ and ‘Blue’ light steps.** Mean ± SEM density of c-Fos expression cells in SCN subregions (**A**) and other major hypothalamic nuclei (**B**) for ‘Yellow’ and ‘Blue’ light pulses (n=6/group). Data analyses by unpaired t-test (ns=P>0.05). MPN: medial preoptic nucleus, AHA: anterior hypothalamic nucleus, LH: lateral hypothalamus, PVN: paraventricular nucleus.


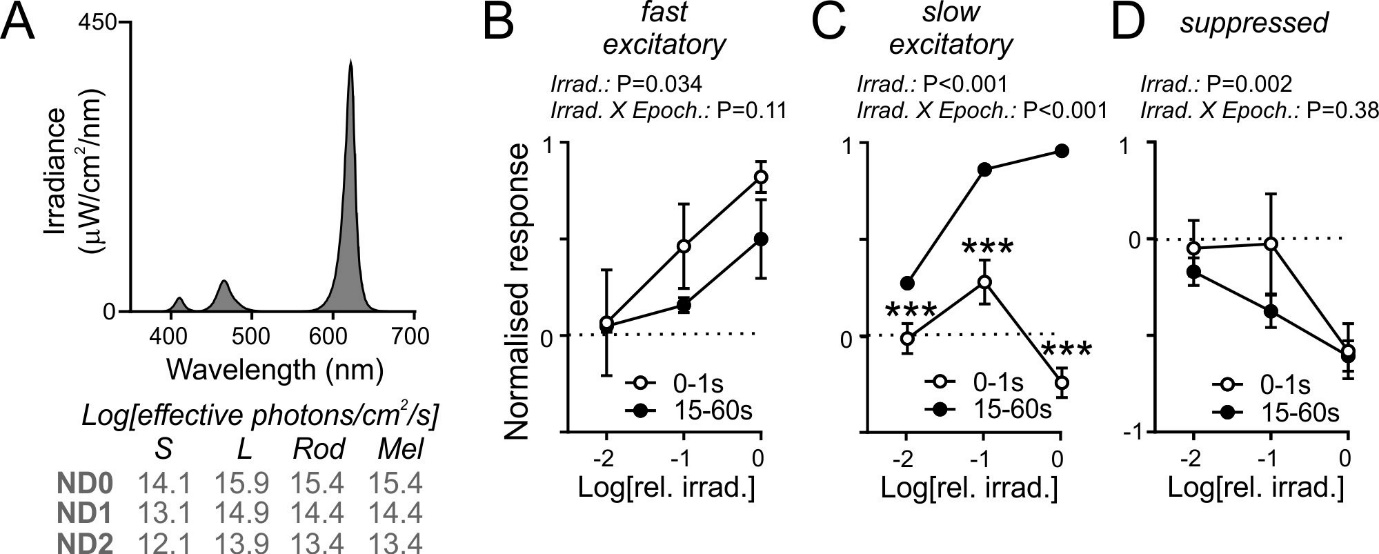


**Figure S5. Analysis of subparaventricular zone sensory responses.** (**A**) Spectral power distribution of the L-cone opsin biased stimulus used to assess irradiance sensitivity in the subparaventricular zone (SPZ) at maximal intensity (left). Right panel shows effective photon flux for *Opn1mw^R^* mouse opsins across the three test intensities for stimuli used in Fig 5A-D. (**B**-**D**) Mean±SEM normalised responses of SPZ cells classified as fast excitatory (**B**; n=5), slow excitatory (**C**; n=21) or light suppressed (**D**; n=20) showing stimulus-evoked change in firing during first 1s and last 45s of 60s light steps from darkness (using stimuli illustrated in **A**). See Additional file 2 for underlying raw data. Data analysed by 2-way RM ANOVA with Sidak’s post-tests (**B**; Irrad.- F_2, 8_= 5.3, P=0.034; Epoch-F_1, 4_= 0.87, P=0.41; Irrad. X Epoch-F_2, 8_=2.97, P=0.11; **C**; Irrad.- F_2, 40_= 28.2, P<0.0001; Epoch-F_1, 20_= 93.2, P<0.0001; Irrad. X Epoch-F_2, 40_=25.9, P<0.0001; **D**; Irrad.- F_2, 38_= 7.6, P=0.002; Epoch-F_1, 19_= 0.97, P=0.34; Irrad. X Epoch-F_2, 28_=1.0, P=0.38).

**
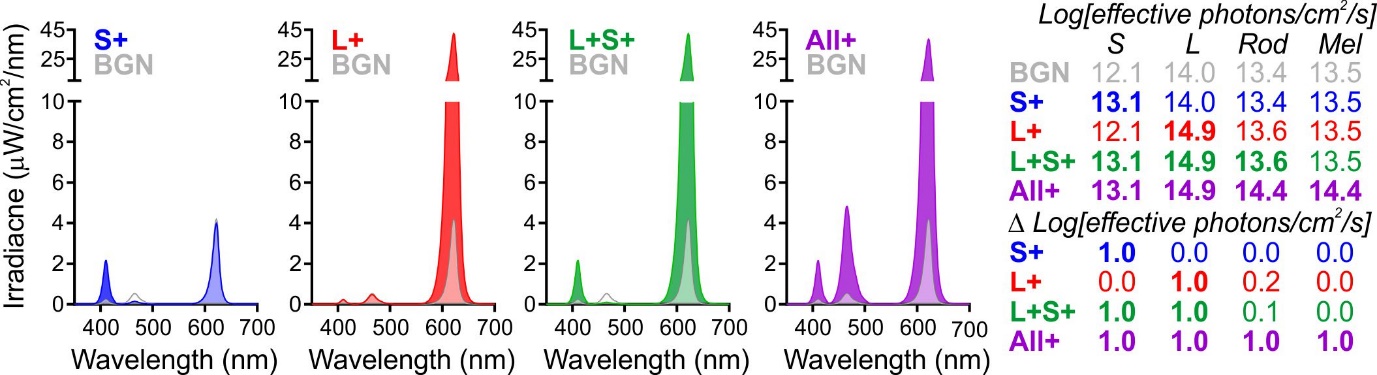
**

**Figure S6. Stimuli for analysing cone influences on subparaventricular neuronal activity.** Left panels show spectral power distributions of test stimuli designed to selectively modulate brightness for S-cone opsin (S+), L-cone opsin (L+), both cone opsins (L+S+), or all opsin classes (All+) relative to a background stimulus (BGN). Right panel shows effective photon flux for all stimuli and change relative to background for stimuli used to generate Fig6A-E.
